# Mortality postponement and compression at older ages in human cohorts

**DOI:** 10.1371/journal.pone.0281752

**Published:** 2023-03-29

**Authors:** David McCarthy, Po-Lin Wang

**Affiliations:** 1 Terry College of Business, University of Georgia, Athens, Georgia, United States of America; 2 Muma College of Business, University of Southern Florida, Tampa, Florida, United States of America; University of Palermo, ITALY

## Abstract

A key but unresolved issue in the study of human mortality at older ages is whether mortality is being compressed (which implies that we may be approaching a maximum limit to the length of life) or postponed (which would imply that we are not). We analyze historical and current population mortality data between ages 50 and 100 by birth cohort in 19 currently-industrialized countries, using a Bayesian technique to surmount cohort censoring caused by survival, to show that while the dominant historical pattern has been one of mortality compression, there have been occasional episodes of mortality postponement. The pattern of postponement and compression across different birth cohorts explain why longevity records have been slow to increase in recent years: we find that cohorts born between around 1900 and 1950 are experiencing historically unprecedented mortality postponement, but are still too young to break longevity records. As these cohorts attain advanced ages in coming decades, longevity records may therefore increase significantly. Our results confirm prior work suggesting that if there is a maximum limit to the human lifespan, we are not yet approaching it.

## Introduction

Whether or not there is a limit to the human lifespan has been a subject of debate for millennia. Historical estimates of the maximum possible lifespan strongly suggest that it has increased substantially over recorded history. The Hebrews of the late Bronze Age famously regarded 80 years as the maximum length of a human life. (Psalm 90:10; <500BC); around 1,000 years later, the ancient Romans set their official estimate of the maximum, the so-called *saeculum naturale*, at 100 or 110 years [1,2]. Modern longevity records are higher still: the current human longevity record is 122, but has remained unchanged since 1997.

Scholarly interest in the topic has not abated [3] use the slow change in longevity records in recent years to argue that the human lifespan has reached an absolute limit, a finding supported by [4,5] use biological hypotheses to reach the same conclusion [6–8], on the other hand, interpret recent improvements in old-age mortality and the pattern of deaths at older ages to assert the opposite.

An important recent study in this area is [9]. They use period data to show that the gap between the percentiles of the distribution of age at death of those older than 65 remained roughly constant between 1960 and 2010 in 20 countries. They conclude that period effects therefore appear to be driving the improvement in longevity at older ages and that lifespan does not appear to be approaching an upper limit.

In this paper, like [9], we analyze mortality of older individuals in richer countries using the Human Mortality Database [10]. However, we analyze mortality by birth cohort, rather than by period. Using cohort data follows a fixed set of individuals over time, and is therefore most suited to clarifying the biological mechanisms underlying mortality. In particular, the use of cohort data may avoid the conflation in period data of changes in mortality rates over time and/or age with changes across cohorts. Cohort analysis is made difficult by cohort censoring due to survival, which we surmount using a Bayesian estimation approach [11–13] that improves the precision of estimates for non-extinct cohorts.

Mortality rates at the highest ages are subject to a great deal of error because the number of people reaching these ages is very small. We therefore approximate mortality by fitting the Gompertz law, which posits that mortality rates in humans increase roughly exponentially with age after around age 50 [14], to each individual cohort between the ages of 50 and 100. As we will demonstrate, the Gompertz law fits cohort mortality data extremely well in this age range, especially in recently extinct cohorts.

We use the Gompertz law to estimate the age (which we call the Gompertzian Maximum Age or GMA) at which individuals first reach an assumed mortality plateau, and test whether this age has changed across birth cohorts or not. If the GMA is found to be constant, this would provide strong evidence in favor of the existence of a maximum limit to human life. In this case, improvements in mortality rates at younger old ages will lead to a compression in the distribution of age at death, called mortality compression. If, however, the GMA increases across cohorts, mortality is being postponed. The presence of mortality postponement would suggest that if there is a maximum limit to human life, it has not yet been reached.

**Fig 1:**
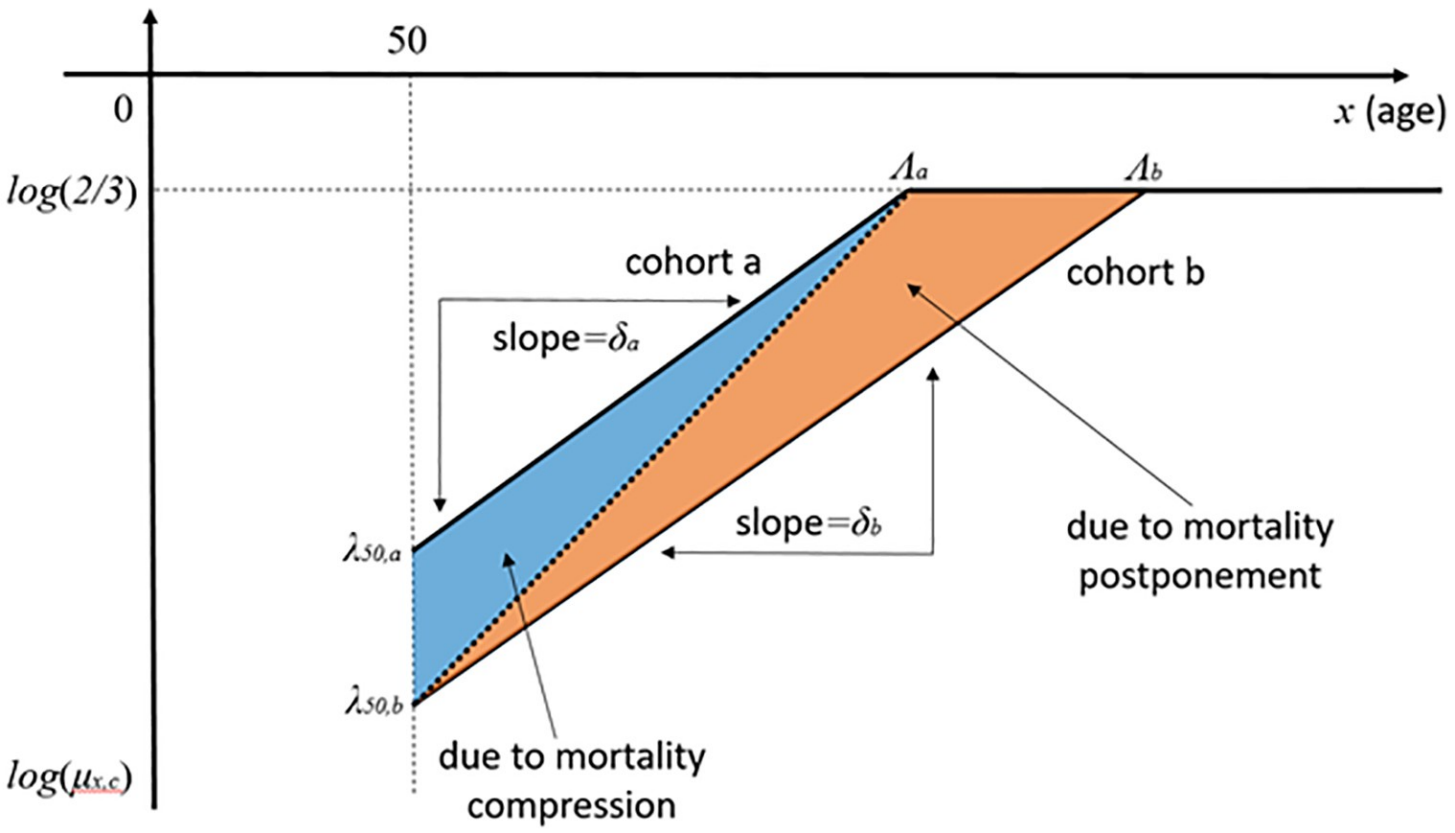
Dividing changes in mortality rates between compression and postponement.

A key parameter in the Gompertz law is the exponential rate of increase in the observed mortality rates with age, called the Rate of Demographic Aging (RDA). Because the RDA determines the distribution of age at death, changes in the RDA across cohorts are a key measure of whether mortality is being postponed or compressed. As shown in Fig 1, if lifespans are approaching an absolute limit, improvements in mortality at younger old ages will be associated with increases in the RDA and the distribution of age at death will be increasingly compressed at advanced ages below the limit. On the other hand if the RDA is constant, improvements in mortality at younger ages will simply shift the distribution of ages at death to the right, suggesting that old-age mortality is being postponed and that a limit to the human lifespan, if it exists, is still far away.

Despite the good fit of the Gompertz law between ages 50 and 100, there are strong theoretical reasons to expect the RDA to fall at extreme old ages. The most likely of these is differential frailty within each cohort [15], leading to selection effects as the frailest individuals in each cohort die earlier, on average, than the less frail. This would cause the RDA to fall gradually at extremely advanced ages, even if the rate of increase of mortality with age of each individual is identical. Such a fall has been observed empirically in the mortality of super-centenarians. In fact, as more high-quality data becomes available, the evidence in support of a levelling-off of the risk of dying has increased [16] found that at extreme ages, mortality probabilities appear to be independent of age and sex, and are around 1/2; [17] suggest that the mortality hazard rate among super-centenarians in Italy appears to level off at around 2/3 (and so annual mortality probabilities are around 1/2), a finding confirmed in other countries by [18].

For analytical convenience, and to be consistent with these findings, we therefore hypothesize the existence of a GMA–the youngest age at which the Gompertz law would predict the mortality hazard rate to be 2/3 –and estimate confidence intervals for this GMA and the age at death of the longest-lived person in each cohort. This approach allows us to disaggregate changes in remaining life expectancy at 50 in historical and current cohorts between postponement and compression explicitly using an analytical method. We emphasize, however, our conclusions on compression and postponement do not depend on either the existence of this plateau or on its assumed level. For instance, if we set the mortality plateau at 1, rather than 2/3, remaining theoretical life expectancy at age 50 would fall by around 5 days, with little variation across cohorts. Removing the plateau altogether causes a fall of a further 4 hours. These values are almost two orders of magnitude smaller than our smallest confidence intervals.

Our analysis reveals that over much of our data, the GMA appears to have remained unchanged, in some countries for centuries. But we do find past and current episodes where mortality postponement has occurred and the GMA has risen. In particular, for cohorts born between 1910 and 1940, we project that the GMA will increase rapidly, confirming the finding of [9] that in recent data, longevity does not appear to be approaching an upper limit. In contrast to this work, however, we show that old-age mortality patterns can be well explained by cohort effects, rather than period effects. These cohort patterns show further why longevity records have not changed in recent decades despite the well-documented improvements in mortality at older ages across much of the industrialized world in recent years. We show that confidence intervals for the length of life of the longest-lived person in each cohort in each country derived using our approach fit historical data on extreme longevity very well. Extrapolating these results forward, we show that it is likely that longevity records will rise as cohorts born after 1910 reach advanced ages, although our projections of by how much they will rise depend on our modelling assumptions.

## Materials and methods

Although more complex approaches are possible, we define the base mortality of individuals aged x and born in year c to be *μ*_*x*,*c*_ and use the Gompertz law to write:

$$\text{log}(\mu_{x,c}^{})=\lambda_{50,c}+\delta_{c}(x-50),50\leq x\leq 100$$ (1)

where *δ*_*c*_ is the RDA of cohort c and λ(_50*c*_) ≡ log(*μ*_50,*c*_). This approach makes the assumption that each cohort is endowed with *δ*_*c*_ and λ_50*c*_ at age 50, and that these remain fixed thereafter. These fixed cohort parameters can be regarded as proxies for causes of death (such as heart disease, stroke, cancer and neurological disorders) that are the consequence of lifestyle and other factors operating over very long time scales. Other than Covid-19, these have become the predominant cause of death among the elderly. We disregard data above age 100 because of evidence that the Gompertz law ceases to apply at these ages, although data here is so sparse that including it in our estimation with appropriate credibility makes little difference. We include data between ages 50 and 80 to ensure that our estimates of the RDA are as precise as possible for each cohort.

To account for shorter-latency causes of death, we allow a cohort’s observed mortality to fluctuate around base mortality due to calendar year effects (e.g. epidemics such as Covid-19 and the 1918 Spanish flu, climatic fluctuations, wars and famines). The error term also captures model misfit (e.g. the gradual fall in the RDA at extreme old age), data errors, and random variation. We therefore write the observed central rate of death at age *x* of an individual born in year *c*, defined as *m*_*x*,*c*_, as:

$$\text{log}(m_{x,c}^{})=\text{log}(\mu_{x,c})+\varepsilon_{x,c}.$$ (2)

We estimated Eq (2) using population mortality data, organized by birth year, for males and females between the ages of 50 and 100 for a set of currently 19 rich industrialized countries (although in some cases our data precede industrialization) from the Human Mortality Database, described in Table 1, along with the set of cohorts used to estimate the three prior distributions we use to test robustness [10].

**Table 1:** Description of data.

	Data period		Extinct cohorts†		Cohorts used to estimate prior distribution			
					Prior A (base case)		Prior B	
Country	Start year	End year	First year of birth	Final year of birth	First year of birth	Final year of birth	First year of birth	Final year of birth
Australia	1921	2017	1871	1917	1871	1927	1871	1927
Austria	1947	2018	1897	1918	1897	1928	1897	1928
Belgium*	1841	2017	1791	1917	1791	1927	1869	1927
Canada	1921	2017	1871	1917	1871	1927	1871	1927
Denmark	1835	2019	1785	1919	1785	1929	1869	1929
Finland	1878	2019	1828	1919	1828	1929	1869	1929
France	1816	2017	1766	1917	1766	1927	1869	1927
Ireland	1950	2016	1900	1916	1900	1926	1900	1926
Italy	1872	2017	1822	1917	1822	1927	1869	1927
Japan	1947	2018	1897	1918	1897	1928	1897	1928
Netherlands	1850	2018	1800	1918	1800	1928	1869	1928
New Zealand	1948	2012	1898	1912	1898	1922	1898	1922
Norway	1846	2019	1796	1919	1796	1929	1869	1929
Portugal	1940	2019	1890	1919	1890	1929	1890	1929
Spain	1908	2017	1858	1917	1858	1927	1869	1927
Sweden	1751	2018	1709	1918	1709	1928	1869	1928
Switzerland	1876	2017	1826	1917	1826	1927	1869	1927
UK	1922	2017	1872	1917	1872	1927	1872	1927
USA	1933	2018	1883	1918	1883	1928	1883	1928

Notes: Population data from the Human Mortality Database was used for each country. Separate estimates were prepared for males and females in each country. † We regard cohorts as extinct when they reach the age of 100.

* Belgian data is incomplete over the war years. Each country’s data is subject to inaccuracies and approximations, especially in earlier years^45^. Prior B only uses period data after 1919 for each country due to concerns about the reliability of earlier data. Prior C uses the same data as Prior B but estimates a single model for all countries (a separate model is used for males and females). All priors use cohorts with data up to age 90, which we define as ‘nearly-estinct’.

### Estimation technique

Details are provided in appendix M1. Combining (1) and (2), the central rate of death at age 50 ≤ *x* ≤ 100 of an individual who was born in year *c*, written *m*_*x*,*c*_, as:

$$\text{log}(m_{x,c}^{})=\lambda_{50,c}+\delta_{c}(x-50)+\varepsilon_{x,c}.$$ (3)

We use changes in the estimates of λ(_50*c*_) and *δ*_*c*_ measured off extinct or nearly-extinct cohorts (where estimation errors are small; nearly-extinct cohorts have data up to age 90) to formulate a Bayesian prior for how these parameters will change over cohorts that are currently censored by survival, and use Bayes’ Theorem to calculate a joint posterior distribution of the parameters for both extinct and censored cohorts conditional on the data and the chosen prior. We allow for fixed-age effects to capture systematic deviations across age from the Gompertz law, such as the slow-down in mortality rates at very high ages. We then use the Metropolis-Hastings (MH) algorithm to draw a sample from this posterior distribution, denoted $\{{\tilde{\lambda }}_{50},\tilde{\delta }\}$, with mode ${\hat{\lambda }}_{50}$ and $\hat{\delta }$.

We then define the Gompertzian Maximum Age of cohort *c* (GMA, denoted Λ_*c*_) as that age at which the base mortality hazard in Eq (1) first reaches 2/3 (so mortality hazards have likely plateaued and annual death probabilities are around 1/2):

$${\hat{\Lambda }}_{c}=\frac{\text{log}(2/3)-{\hat{\lambda }}_{50,c}}{{\hat{\delta }}_{c}}+50$$ (4)

### Division between compression and postponement

Details are provided in appendix M2. We used our MH sample $\{{\tilde{\lambda }}_{50},\tilde{\delta }\}$ to quantify the changes in remaining cohort base life expectancy at age 50 between cohorts born ten years apart due to compression and postponement, as well as 95% confidence intervals. As shown in Fig 1, the full change in λ_50,*c*_ and any consequent change in *δ*_*c*_ needed to keep Λ_*c*_ fixed were ascribed to compression, and any further changes in *δ*_*c*_ to postponement.

### 95% confidence intervals for the GMA and the maximum length of life

Details are provided in appendix M3. We then calculated 95% confidence intervals for Λ_*c*_ directly using our sample $\{{\tilde{\lambda }}_{50},\tilde{\delta }\}$ and (4), and confidence intervals for *M*_*c*_, the age at death of the longest-lived person in each cohort conditional on at least one person in each cohort reaching age Λ_*c*_, under the assumption that the mortality hazard at ages older than Λ_*c*_ (so after the plateau) are constant and equal to two thirds. Although Λ_*c*_ only changes with underlying mortality rates, *M*_*c*_ will increase as cohort size increases even if mortality rates remain the same.

## Results

**Fig 2:**
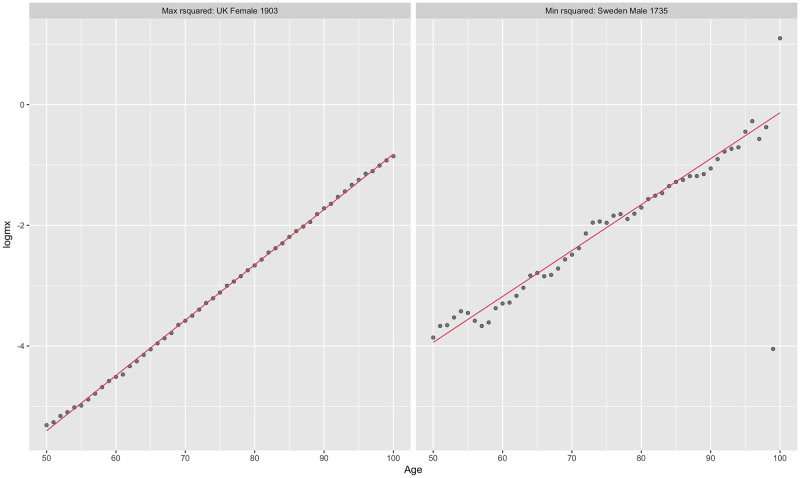
Extinct cohorts with highest (left) and lowest (right) R-squared in our dataset.

**Fig 3:**
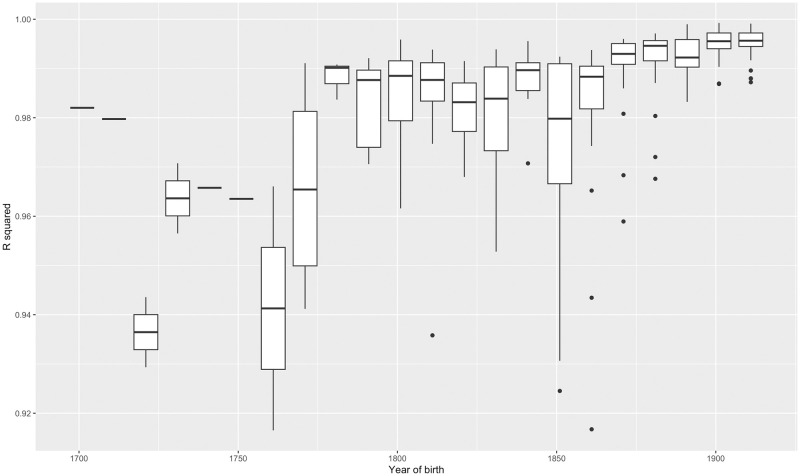
Box-and-whisker plot of the distribution R-squared across extinct cohorts, by decade of birth.

**Fig 4:**
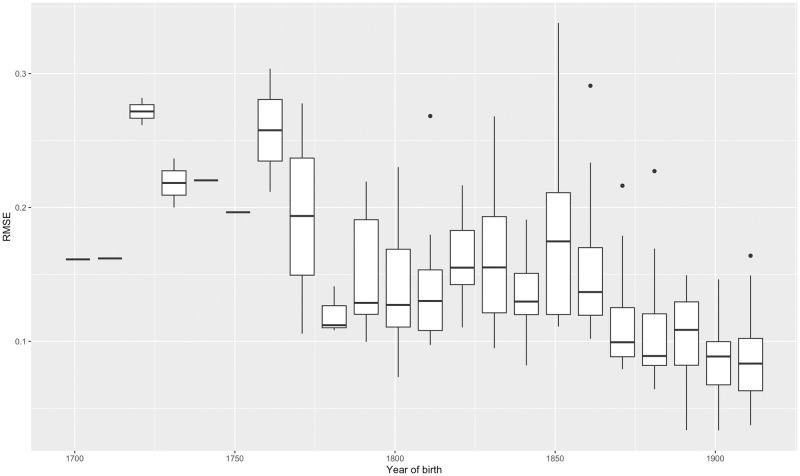
Box-and-whisker plot of the distribution of the RMSE across extinct cohorts, by decade of birth.

Despite its simplicity, the Gompertz law accounts for the vast majority of the variation in observed mortality rates between ages 50 and 100 across the extinct cohorts in our data (here we regard cohorts as extinct when they have reached the age of 100). Fig 2 shows the extinct cohorts with the highest and lowest R-squared values. Using ${\hat{\lambda }}_{50}$ and $\hat{\delta }$ as point estimates, the modal value of R-squared of (2) for extinct cohorts is around 0.9925 (0.9975 for the most recent extinct cohorts, born in the 1910’s). The residual mean squared error (RMSE) of (2), which represents the average percentage difference between actual mortality rates and estimated base mortality rates due to all causes declines from around 20% of mortality rates for the very earliest extinct cohorts to around 7% of mortality rates for the latest ones, born in the 1910’s. Fig 3 shows the increase in R-squared over extinct cohorts; while Fig 4 shows the decline in RMSE.

The main source of differences driving the results in Figs 3 and 4 are calendar year effects representing short-latency causes of death. Examples are the 1918 influenza epidemic and the Covid-19 epidemic, which caused the mortality of affected cohorts to rise at the age they were when the epidemics occurred. Other examples might be climatic fluctuations, wars or famines. In general, calendar year effects are more significant for earlier cohorts of our sample, but have fallen in magnitude following World War II, as a result of greater prosperity, technological advancements in living standards and healthcare, and increased political and economic stability in the countries we examine [19,20], although Covid-19 represents a significant exception. A small increase in the RMSE for cohorts born around 1850 is likely attributable to the Spanish Flu epidemic of 1918 and the First World War, and an increase for cohorts born around 1890 to the Second World War.

A second source of error is model misfit, as the Gompertz law may not fully capture the underlying dynamics of mortality. As discussed, the RDA may fall at extreme ages (which we capture to some extent through the use of age fixed-effects), senescent aging in females may be delayed due to childbirth, and calendar-year effects may have significant autocorrelation (a factor we ignore in our estimation procedure).

Random variation is especially important at older ages where relatively few cohort members are still alive, which we account for by ascribing less credibility to ages at which fewer deaths occur [21].

A final source of error is data errors. With some caveats, data for recent years are likely to be accurate and substantially complete in all the countries we examine. However, in earlier years (before World War II in particular), data may be smoothed or imputed from periodic censuses or grouped data, or may simply be inaccurate. Readers can consult the data appendices for each country for more information about data errors when interpreting our results [10].

**Fig 5:**
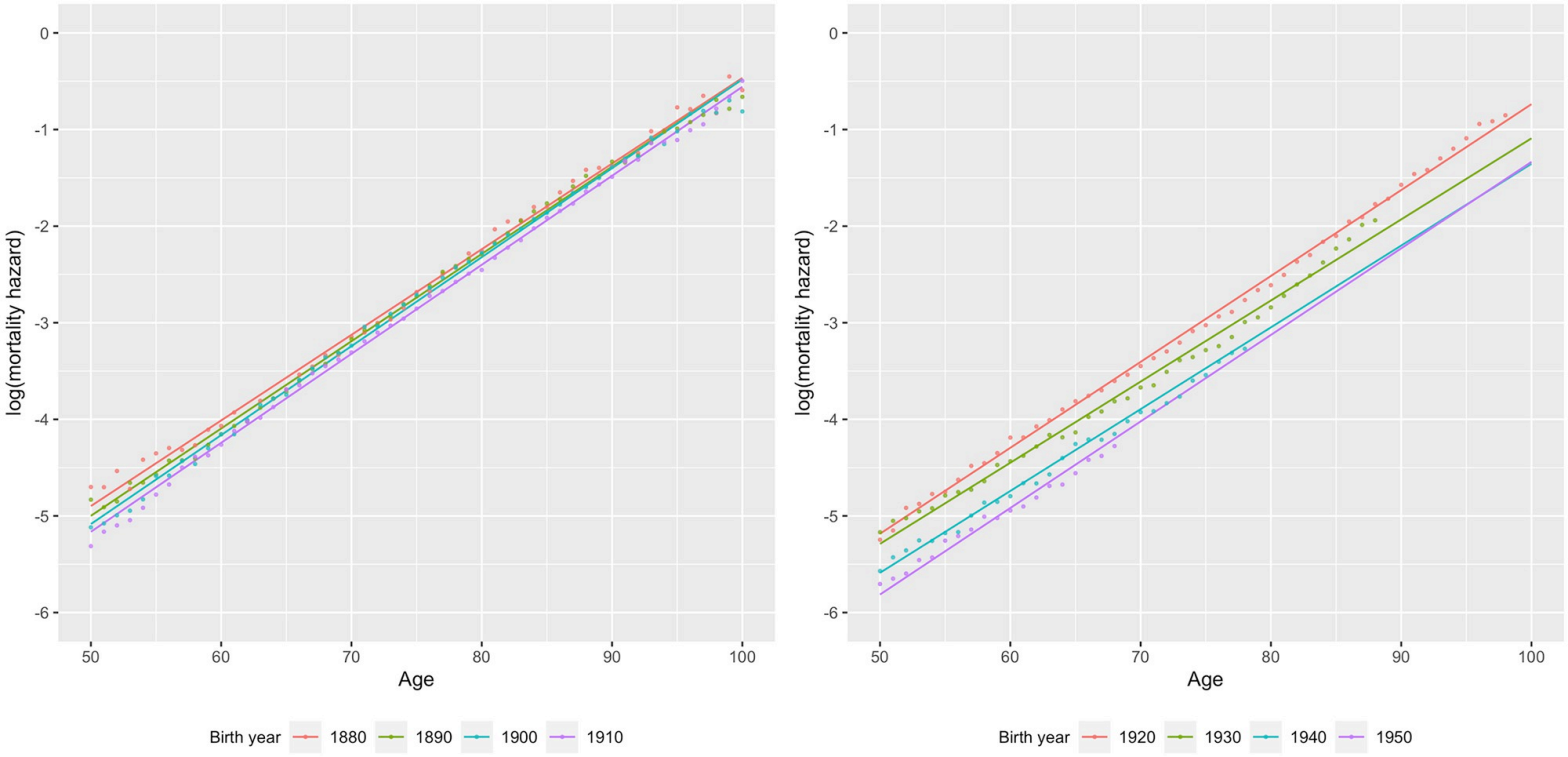
Observed mortality hazard rates (dots) and estimated base mortality hazard rates (lines) by age for Swedish males born 1880–1950. Note: Estimated base mortality rates are calculated using modal estimates ${\hat{\lambda }}_{50,c}$ and ${\hat{\delta }}_{c}$ for each cohort.

Because Sweden has the longest data series in our sample, and exhibits patterns that are widely similar to other countries, we focus on results from Sweden before turning to other countries. Fig 5 shows log observed mortality hazard rates by age for Swedish males for cohorts born ten years apart from 1880 to 1950 (dots), along with our estimates of log base mortality (lines, calculated using ${\hat{\lambda }}_{50,c}$ and ${\hat{\delta }}_{c}$). As shown in the left panel, ${\hat{\lambda }}_{50,c}$ fell slightly for cohorts born between 1880 and 1910, but because ${\hat{\delta }}_{c}$ rose to compensate, there was little change in mortality rates at very old ages, consistent with mortality compression. Cohorts born after 1910, shown in the right panel, exhibit a strikingly different pattern: declines in ${\hat{\lambda }}_{50,c}$ were larger, but there appears to be little evidence of an increase in ${\hat{\delta }}_{c}$, a pattern strongly consistent with mortality postponement. Unless there is a dramatic increase in their RDA’s only at older ages, a pattern for which there appears to be little or no historical precedent, we can expect that the GMA and the maximum age at death of individuals in these cohorts will rise.

**Fig 6:**
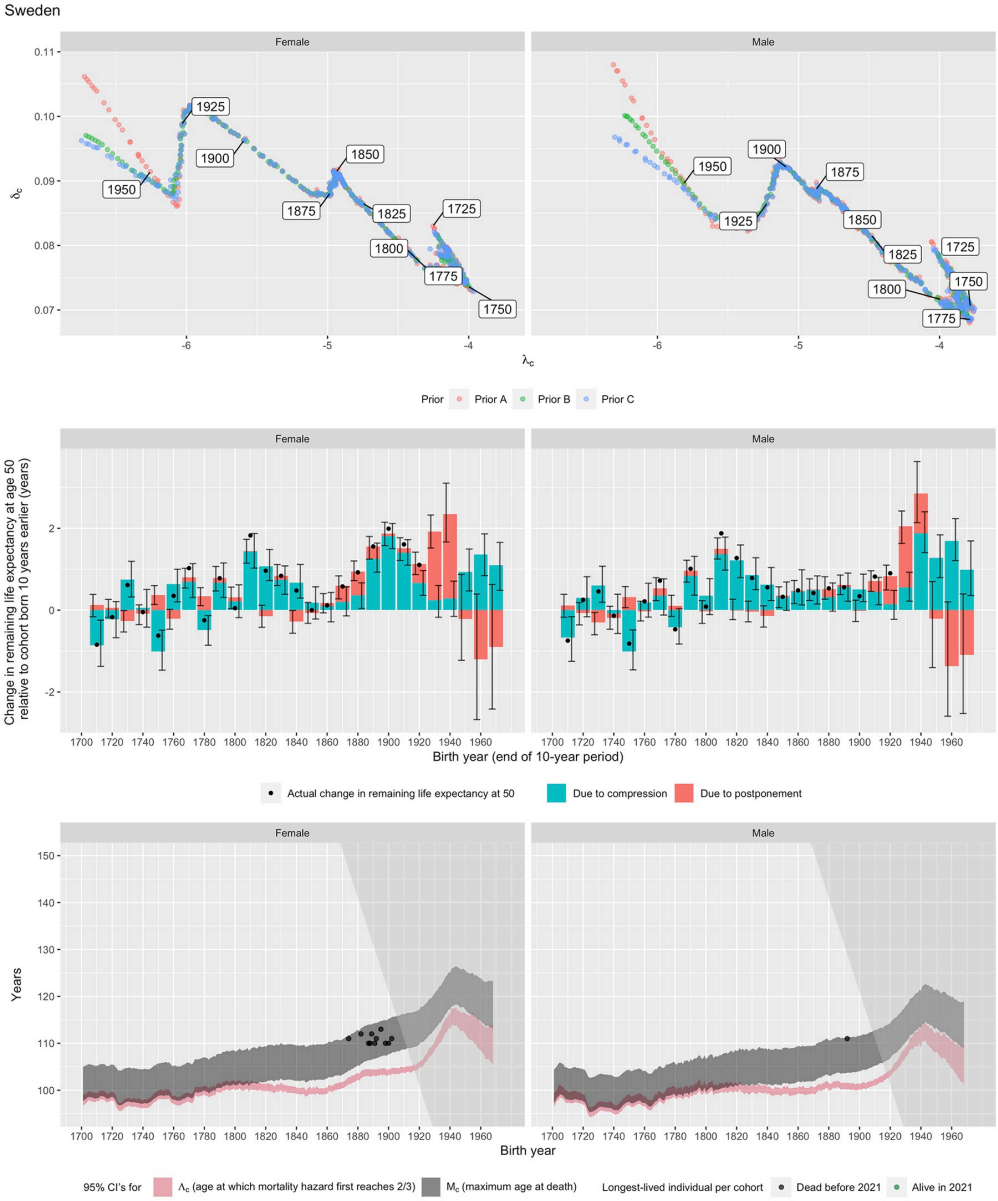
Bayesian estimates of slope and intercept parameters of male and female cohorts for Sweden (panel A); implied changes in remaining cohort base life expectancy at age 50 over ten year birth cohorts, divided into the portion due to postponement and portion due to compression (panel B), and 95% confidence intervals for the age at which cohort mortality hazard first reaches 2/3 (Λ_*c*_) and the maximum observed lifespan (*M*_*c*_) in each birth cohort (panel C). Note: Confidence intervals are not shown in Panel A for ease of interpretation. In the other panels, bands or bars show 95% confidence intervals for the true base value. In panel B, bar heights show median estimates. Black dots show the actual change in life expectancy for extinct cohorts. Note that under the assumption that the model is correct, this would equal the true base value plus random error due to calendar-year effects and random variation, plus the change in bias due to these. In panel C, black dots show the longest-lived person in each birth cohort from IDL [22] and GRG [23] data (green if they are still alive in 2021). The mean of *M*_*c*_ increases in line with the logarithm of cohort size at age 50 even if there are no changes in mortality rates. The shaded area to the right of the diagonal line in panel C indicates censoring in 2021.

Fig 6 shows full results for Swedish females (left column) and males (right column). Panel A shows ${\hat{\lambda }}_{50,c}$ plotted against ${\hat{\delta }}_{c}$ for males and females by birth cohort. Females appear to have lower values of ${\hat{\lambda }}_{50,c}$ but higher values of ${\hat{\delta }}_{c}$ than males of the same cohort. ${\hat{\delta }}_{c}$ varies between 0.07 and 0.10 per year of age, and is not obviously constant across cohorts. In fact, statistical tests based on the Bayesian Information Criterion (BIC) and the Akaike Information Criterion (AIC), shown for all countries in Table 2, decisively reject the hypothesis that *δ*_*c*_ is constant across cohorts in Sweden for both males and females. Although changes in ${\hat{\lambda }}_{50,c}$ and ${\hat{\delta }}_{c}$ are small and appear random for the earliest cohorts (which may be related to data issues (see the data appendix for Sweden at HMD, 2021)), starting with cohorts born around 1775 the dominant pattern is that falls in ${\hat{\lambda }}_{50,c}$ are associated with rises in ${\hat{\delta }}_{c}$ for both males and females, a pattern associated with mortality compression (similar to Panel A of Fig 5). There are, however, two sets of cohorts for which both ${\hat{\delta }}_{c}$ and ${\hat{\lambda }}_{50,c}$ fell, a pattern associated with mortality postponement. The first occurred for cohorts born roughly between 1855 and 1875 for females (later and shorter for males), but was of much shorter duration and lower significance than the second such period, illustrated in Panel B of Fig 5, which is projected to occur largely for cohorts that are still censored by survival: those born between around 1920 and 1945 for females (1902 and 1930 for males). The historically dominant pattern of increases in ${\hat{\delta }}_{c}$ but falls in ${\hat{\lambda }}_{50,c}$ is expected to resume for cohorts born after about 1945 (although these estimates are imprecise and depend heavily on the chosen prior).

**Table 2:** Statistical tests for *δ*_*c*_ = *δ ∀ c*.

	Males				Females			
	AIC		BIC		AIC		BIC	
Country	Constant *δ*	Variable *δ*_*c*_	Constant *δ*	Variable *δ*_*c*_	Constant *δ*	Variable *δ*_*c*_	Constant *δ*	Variable *δ*_*c*_
Australia	-14,448	-14,796	-13,827	-13,579	-14,424	-14,520	-13,803	-13,303
Austria	-9,168	-9,928	-8,734	-9,083	-9,520	-9,618	-9,086	-8,774
Belgium	-29,044	-29,784	-27,799	-27,323	-26,869	-30,204	-25,624	-27,742
Canada	-17,222	-18,000	-16,601	-16,784	-14,789	-17,497	-14,168	-16,280
Denmark	-26,109	-29,494	-24,792	-26,888	-28,076	-32,015	-26,758	-29,408
Finland	-19,356	-20,235	-18,387	-18,324	-19,335	-20,685	-18,365	-18,771
France	-32,791	-35,427	-31,334	-32,541	-30,019	-33,769	-28,561	-30,883
Ireland	-7,602	-8,818	-7,205	-8,047	-8,353	-8,450	-7,956	-7,679
Italy	-22,652	-23,630	-21,650	-21,652	-19,733	-22,175	-18,731	-20,197
Japan	-10,456	-12,811	-10,023	-11,967	-9,517	-11,105	-9,083	-10,261
Netherlands	-23,370	-26,309	-22,192	-23,979	-21,722	-27,637	-20,543	-25,306
New Zealand	-7,080	-7,729	-6,698	-6,987	-7,305	-7,541	-6,923	-6,799
Norway	-25,500	-27,785	-24,272	-25,358	-23,539	-29,961	-22,312	-27,533
Portugal	-10,733	-11,549	-10,240	-10,587	-9,959	-10,765	-9,466	-9,802
Spain	-11,915	-14,302	-11,194	-12,887	-9,609	-12,806	-8,889	-11,391
Sweden	-33,316	-40,311	-31,306	-36,320	-32,313	-40,588	-30,301	-36,593
Switzerland	-22,705	-24,075	-21,734	-22,161	-17,909	-21,350	-16,939	-19,435
UK	-16,230	-17,525	-15,617	-16,323	-16,236	-18,319	-15,623	-17,118
USA	-15,463	-16,048	-14,925	-14,997	-13,035	-15,896	-12,497	-14,844

Note: The AIC and BIC prefer the model with the lowest criterion. In each case, the preferred model is highlighted in grey.

Panel B of Fig 6 illustrates our median estimates of the change in remaining base life expectancy at age 50 for cohorts born ten years apart, split into the portions due to compression and postponement, with 95% confidence intervals for each component. The pattern of postponement and compression described previously, and strikingly evident in Fig 5, is clear. We project that Swedish females born in 1950 can expect to live 7.5 years (males: 9.5 years) longer after the age of 50 than their counterparts born 50 years earlier. 60% (males: 70%) of this increase will be due to postponement. Postponement, but not compression, is projected to end for cohorts born after 1950 (although again these estimates are highly imprecise). Black dots in panel B show the actual change in cohort life expectancy over ten-year periods for extinct cohorts. These are close to our median estimates, but always lie within our overall confidence intervals.

Panel C shows confidence intervals (in red) for Λ_*c*_, the Gompertzian maximum age at which the mortality hazard rate first reaches two thirds. Remarkably, Λ_*c*_ barely changed for males born over the two centuries before 1900 (or for females born between 1700 and 1860, after which the first period of mortality postponement identified in Panel A increased Λ_*c*_ by around 5 years), confirming that mortality postponement was the dominant pattern for these cohorts. Equally remarkably, despite different values of ${\hat{\delta }}_{c}$ and ${\hat{\lambda }}_{50,c}$ for males and females, Λ_*c*_ was very similar for Swedish males and females born between 1700 and 1860.

Panel C also shows 95% confidence intervals (in grey) for *M*_*c*_, the age at death of the longest-lived person in each cohort, conditional on at least one person in each cohort reaching age Λ_*c*_. Where data is available in the International Database on Longevity (IDL [22]) and a database maintained by the Gerontology Research Group (GRG [23]), we plotted the actual age at death of the longest-surviving male and female in each birth cohort. The shading indicates the age/year-of-birth combinations that are censored by survival, with the darker shade to the right representing censoring. For cohorts born before 1900, the mean of *M*_*c*_ increases due to increases in population, mortality compression and small amounts of postponement. But for cohorts born after 1900, the dramatic period of mortality postponement identified in Panel B may raise the observed maximum lifespan significantly in addition to the increases caused by population change. For instance, our estimates indicate that there is a 95% chance that the last Swedish female born in 1950 will only die aged between 117.1 and 125.5 years (so sometime between 2067 and 2075), assuming that the force of mortality plateaus at 2/3.

**Fig 7:**
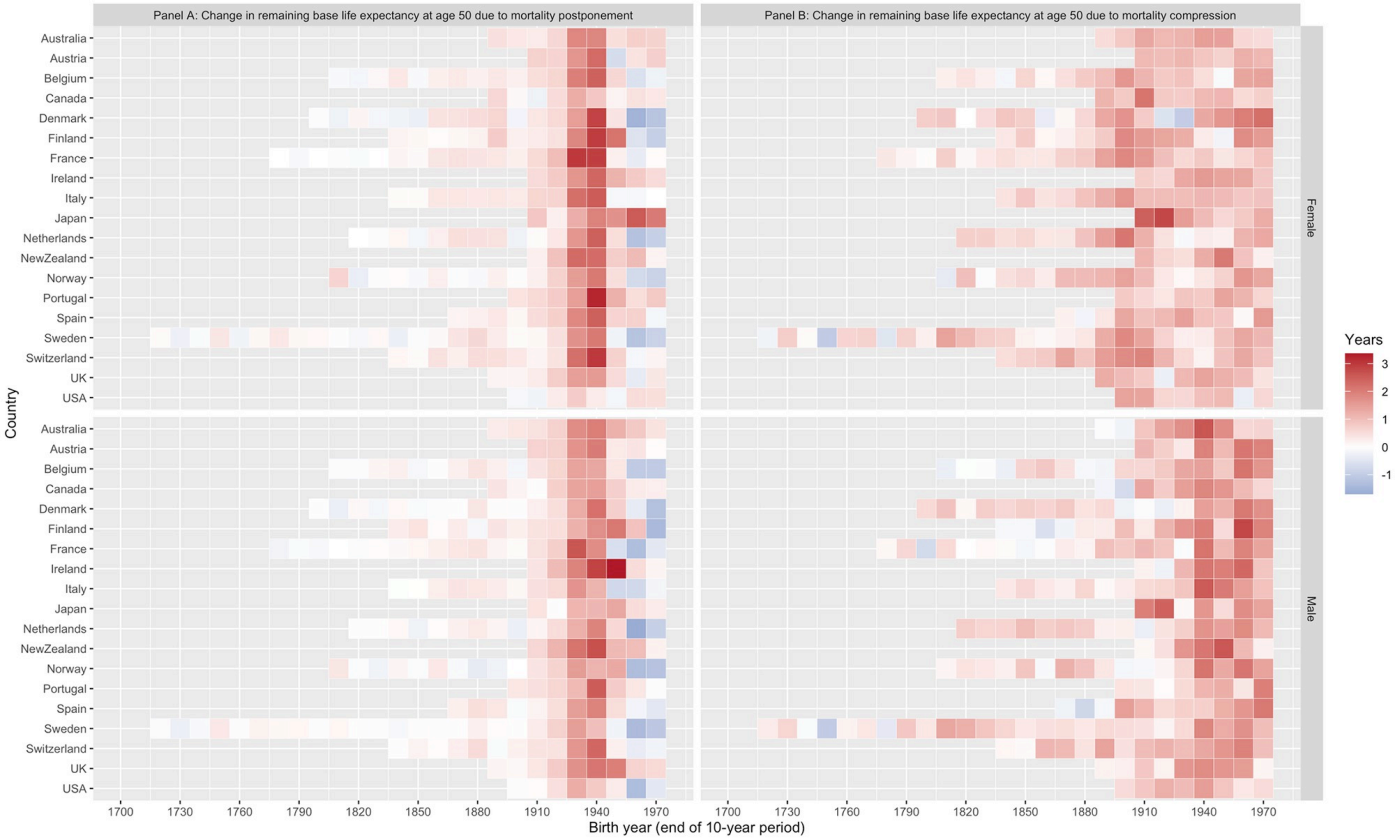
Changes in remaining base life expectancy at age 50 across 10-year birth cohorts separated into the portion due to postponement and the portion due to compression for males and females. Note: These show the median changes in remaining cohort life expectancy at age 50 due to compression and postponement estimated from our posterior sample $\left\{{\tilde{\lambda }}_{50},\tilde{\delta }\right\}$ for each country. Confidence bands for earlier cohorts are small, but widen for the most recent two decades shown. Detailed results for each country, along with confidence intervals, are available in the supplementary materials.

Fig 7 summarizes our median estimates for all 19 countries of the changes in remaining cohort life expectancy at age 50 between cohorts born 10 years apart, split between compression and postponement. Compression is the dominant pattern for cohorts born before 1900, replaced by postponement for those born after. These results show clearly why the distribution of the maximum lifespan has changed so little: cohorts that could have reached the maximum lifespan of 122 before 2021 were born before 1899, and so experienced improvements in life expectancy that were driven largely by compression.

**Fig 8:**
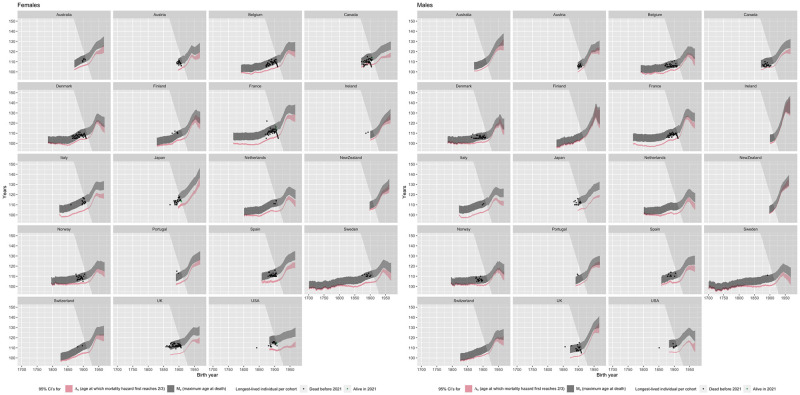
95% confidence intervals for the length of maximum life (*M*_*c*_, grey) and the Gompertzian maximum age (Λ_*c*_, red). Note: Dots in the figures show the age at death (black dots) and current age (green triangles) of the longest-lived person in each birth cohort, from the IDL [22] and the GRG [23]. IDL data contains people over the age of 105 or 110 (depending on country); GRG data only show people over the age of 110. The mean length of maximum life increases due to compression and population increase (because more people are expected to reach age Λ_*c*_) as well as postponement. Confidence intervals for *M*_*c*_ are conditional on at least one person reaching age Λ_*c*_. Unconditional upper confidence intervals for *M*_*c*_ are always the same as the conditional ones, but the unconditional lower confidence interval may be smaller than shown in countries with small populations, especially for males. See methods section M3 for details.

Observed mortality patterns of censored cohorts suggest that this is likely to change as the cohorts that are benefiting from mortality postponement reach extreme old age. Fig 8 shows our estimated 95% confidence intervals for the GMA and the maximum observed lifespan of males and females in each country for cohorts born before 1970. (Figures similar to Fig 8, but showing Panels A and B of Fig 5 are shown for all countries in S1 File) The height of the confidence intervals depend on our assumption that the mortality hazard rate plateaus at 2/3. The fact that our confidence intervals for *M*_*c*_ fit IDL and GRG data very well in almost all countries provides further evidence that our assumed mortality plateau of 2/3 is not an unreasonable assumption, at least in past cohorts.

**Fig 9:**
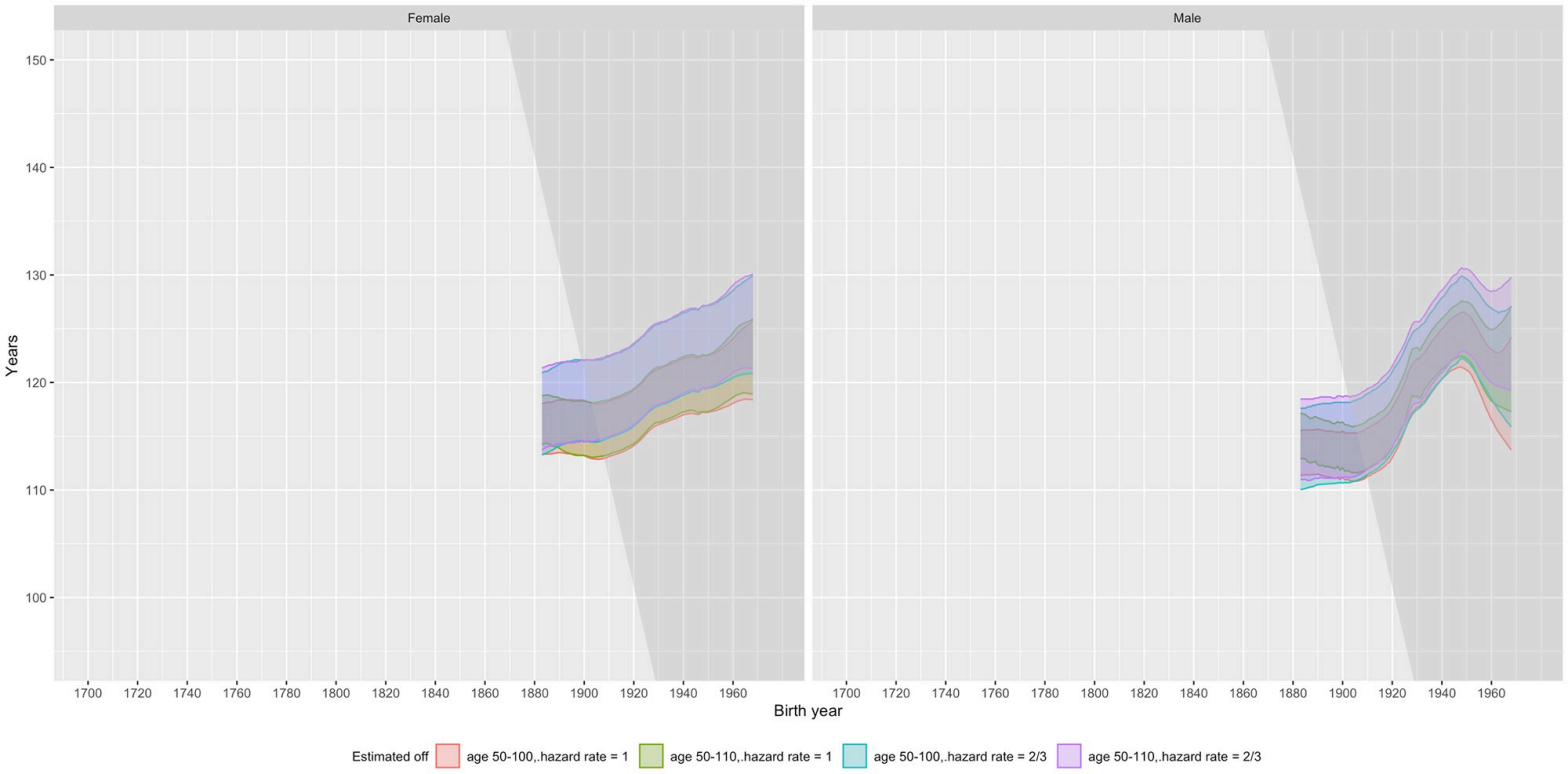
Confidence intervals for *M*_*c*_ for the USA using combinations of use of data ages and assumption of the height of the plateau.

But we caution that our predictions of the length of life of the longest-lived individuals–based on extrapolating past patterns into the future–do depend on this assumption. The higher the assumed level of the mortality plateau, the lower the level of these confidence intervals will be. For instance, Fig 9 shows our estimated confidence intervals for the US under different assumptions of the value of the threshold and different data (50–100 vs 50–110). The data set makes little difference, but raising the height of the plateau to 1 from 2/3 reduces the height of the confidence intervals by as much as 4 years.

However, even if we assume a much higher mortality plateau than data on past longevity record holders suggests would be appropriate, our results do show clearly that significant potential exists for longevity records to be broken in country after country and globally as cohorts born in the 1930’s and 1940’s reach extreme old age. Japanese females–who are already close to reaching longevity records–are simply at the forefront of this phenomenon.

As in all Bayesian approaches, our choice of prior is subjective. We therefore repeated our results for the three choices of prior shown in Table 1. Results are shown in the appendix. We found that our estimates of postponement are robust to the choice of prior for cohorts born before around 1950, indicating a very strong signal from the data for these cohorts that mortality postponement is indeed occurring. We therefore emphasize results only for cohorts born before 1950. We also back-tested the model for Swedish data, and found reasonable, but not perfect, out-of-sample predictive power. Results are shown in S1 File.

## Discussion

We summarize historical mortality data in 19 currently-industrialized countries by birth cohort using a variant of the Gompertz mortality law, and find that it fits cohort mortality data extremely well. Using this law, we identify the youngest age at which individuals in each cohort reach an assumed mortality plateau, which we call the Gompertzian Maximum Age (GMA). We find that over much of the period covered by our data, there was no increase in the GMA. Historical improvements in life expectancy were therefore largely the result of mortality compression. We demonstrate, however, that there have been episodes where the GMA increased. The presence of these episodes of mortality postponement suggests that the maximum length of a human life is not, in fact, fixed.

The first episode of mortality postponement that we identify occurred for cohorts born in the early part of the second half of the 19^th^ century, and was more pronounced for females than for males. Over this period, the GMA increased by around 5 years. We can only speculate as to the causes of this increase, but as the first of these cohorts reached age 50 just after 1900 and the last reached age 100 in 1980, this may be related to a first wave of improvements in public health and medical technology.

We identify a second, and much more significant, episode of mortality postponement, which is affecting cohorts born between 1910 and 1950 (so those currently aged between 70 and 110). We estimate that the GMA for these cohorts may increase by as much as 10 years, and remaining life expectancy at age 50 by as much as 8 years, depending on the country.

The timing of these episodes of mortality postponement explain why longevity records have been so slow to increase in recent years–cohorts old enough to have broken longevity records were too old to experience the current bout of postponement–and identifies significant potential for longevity records to rise by the year 2060 as younger cohorts, who did experience it, reach advanced old age.

Our results on the division of changes in remaining life expectancy at age 50 across cohorts between compression and postponement are robust to our modelling choices. Likewise, our conclusion that longevity records will likely be broken in the coming decades is also robust to a wide range of possible assumptions. But our predictions of precisely by how much these records will rise, and when, depend on our modelling assumptions, in particular on the maximum mortality rate we assume.

We emphasise further that cohorts born before 1950 will only have the potential to break existing longevity records if policy choices continue to support the health and welfare of the elderly, and the political, environmental and economic environment remains stable. The emergence of Covid-19 and its outsize effect on the mortality of the elderly provides a salutary warning that none of this is certain. If, however, the GMA does increase as the current mortality experience of incomplete cohorts suggests is likely, the implications for human societies, national economies and individual lives will be profound.

## Supporting information

S1 Fig Bayesian estimates of slope and intercept parameters of male and female cohorts for all countries. Note: The points show the modal estimate of the RDA (*δ*_*c*_) and logged mortality rates at age 50 (λ_*c*_) for each cohort. Confidence intervals are not shown to increase clarity.
(TIF)

S2 Fig Implied changes in remaining cohort base life expectancy at age 50 over ten-year birth cohorts, divided into the portion due to postponement and portion due to compression. Note: The coloured bars show the median estimate of 10-year changes in remaining cohort life expectancy at age 50, divided into the portion due to postponement and the portion due to compression, determined using the methodology shown in Fig 1 of the main text and formulae derived in the methods section. Black dots represent the actual change in life expectancy for cohorts that reached the age of 100 in our data.
(TIF)

S3 Fig Results for Prior B. (TIF)

S4 Fig Results for Prior C. (TIF)

S5 Fig Backtesting for Sweden. Confidence intervals for Λ_*c*_ data to 1900, data to 1950 and all data.
(TIF)

S6 Fig Backtesting for Sweden. Confidence intervals for *M*_*c*_ using data to 1900, data to 1950 and all data.
(TIF)

S7 Fig Backtesting for Sweden. Predicted changes in base life expectancy due to compression and postponement using all data to 1900, data to 1950 and all data.
(TIF)

S8 Fig Bayesian estimates of slope and intercept parameters of male and female cohorts for all countries using data ages 50–100 v 50–110. (TIF)

S9 Fig Difference between estimates of changes in life expectancy due to compression and postponement for Sweden when data from 50–110 is used relative to when data from 50–100 is used. (TIF)

S10 Fig Difference between estimates of changes in life expectancy due to compression and postponement for Sweden when mortality plateau is assumed to be 1 relative to when profile is assumed to be 2/3 (data from 50–100). (TIF)

S11 Fig Difference between estimates of changes in life expectancy due to compression and postponement for Sweden when mortality plateau is assumed to be 1 and when data from 50–110 is used relative to when profile is assumed to be 2/3 and data from 50–100 is used. (TIF)

S12 Fig Difference between estimates of changes in life expectancy due to compression and postponement for all countries when data from 50–110 is used relative to when data from 50–100 is used. (TIF)

S13 Fig Difference between estimates of changes in life expectancy due to compression and postponement for all countries when mortality plateau is assumed to be 1 relative to when profile is assumed to be 2/3 (data from 50–100). (TIF)

S14 Fig Difference between estimates of changes in life expectancy due to compression and postponement for all countries when mortality plateau is assumed to be 1 and when data from 50–110 is used relative to when profile is assumed to be 2/3 and data from 50–100 is used. (TIF)

S1 File (DOCX)

S2 File (DOCX)

## References

[1] Psalm 90:10, King James Bible (date unknown, likely before 500BC/1611).

[2] Horace, Carmen Saeculare, ed. Fr. Vollmer, Teubner, Leipzig (17BC/1917), stanza VI, English text available here https://www.poetryintranslation.com/PITBR/Latin/HoraceEpodesAndCarmenSaeculare.php#anchor_Toc98670048.

[3] Dong X, Milholland B, Vijg J. Evidence for a limit to human lifespan. *Nature*. 2016 Oct 13;538(7624):257–9. doi: 10.1038/nature19793 27706136 PMC11673931

[4] Olshansky SJ. Measuring our narrow strip of life. *Nature*. 2016 Oct 13;538(7624):175–6. 27706138 10.1038/nature19475

[5] Kirkwood TB, Austad SN. Why do we age?. *Nature*. 2000 Nov 9;408(6809):233–8. doi: 10.1038/35041682 11089980

[6] Oeppen J, Vaupel JW. Broken limits to life expectancy. *Science*. 2002 May 10;296(5570):1029–31. 12004104 10.1126/science.1069675

[7] Vaupel JW. Biodemography of human ageing. *Nature*. 2010 Mar 25;464(7288):536–42. doi: 10.1038/nature08984 20336136 PMC4010874

[8] Rootzén H, Zholud D. Human life is unlimited–but short. *Extremes*. 2017 Dec;20(4):713–28.

[9] Zuo W, Jiang S, Guo Z, Feldman MW, Tuljapurkar S. Advancing front of old-age human survival. *Proceedings of the National Academy of Sciences*. 2018 Oct 30;115(44):11209–14. doi: 10.1073/pnas.1812337115 30327342 PMC6217443

[10] Human Mortality Database www.mortality.org (accessed 2021).

[11] McCarthy D. 80 will be the new 70: Old‐age mortality postponement in the United States and its likely effect on the finances of the OASI program. *Journal of Risk and Insurance*. 2021 Jun;88(2):381–412.

[12] McCarthy D, Wang PL. An analysis of period and cohort mortality shocks in international data. *North American Actuarial Journal*. 2021 Feb 18;25(sup1):S385–409.

[13] McCarthy DG, Wang PL. Pooling mortality risk in Eurozone state pension liabilities: An application of a Bayesian coherent multi-population cohort-based mortality model. *Insurance*: *Mathematics and Economics*. 2021 Jul 1;99:459–85.

[14] Gompertz B. XXIV. On the nature of the function expressive of the law of human mortality, and on a new mode of determining the value of life contingencies. In a letter to Francis Baily, Esq. FRS &c. *Philosophical transactions of the Royal Society of London*. 1825 Dec 31(115):513–83.

[15] Vaupel JW, Manton KG, Stallard E. The impact of heterogeneity in individual frailty on the dynamics of mortality. *Demography*. 1979 Aug;16(3):439–54. 510638

[16] Rootzén H, Zholud D. Human life is unlimited–but short. *Extremes*. 2017 Dec;20(4):713–28.

[17] Barbi E, Lagona F, Marsili M, Vaupel JW, Wachter KW. The plateau of human mortality: Demography of longevity pioneers. *Science*. 2018 Jun 29;360(6396):1459–61. doi: 10.1126/science.aat3119 29954979 PMC6457902

[18] Alvarez JA, Villavicencio F, Strozza C, Camarda CG. Regularities in human mortality after age 105. *PloS one*. 2021 Jul 14;16(7):e0253940. doi: 10.1371/journal.pone.0253940 34260647 PMC8279393

[19] Carson C, Hajat S, Armstrong B, Wilkinson P. Declining vulnerability to temperature-related mortality in London over the 20th century. *American journal of epidemiology*. 2006 Jul 1;164(1):77–84. doi: 10.1093/aje/kwj147 16624968

[20] Åström DO, Forsberg B, Edvinsson S, Rocklöv J. Acute fatal effects of short-lasting extreme temperatures in Stockholm, Sweden: evidence across a century of change. *Epidemiology*. 2013 Nov 1;24(6):820–9. doi: 10.1097/01.ede.0000434530.62353.0b 24051892

[21] Fay RE III, Herriot RA. Estimates of income for small places: an application of James-Stein procedures to census data. *Journal of the American Statistical Association*. 1979 Jun 1;74(366a):269–77.

[22] International Database on Longevity www.supercentenarians.org (accessed 2021).

[23] . Gerontology Research Group grg.org (accessed 2021).

