# Supplementary figures and images for: Mortality postponement and compression at older ages in human cohorts
Source: PLoS One. 2023 Mar 29;18(3):e0281752. doi: 10.1371/journal.pone.0281752 (PMC10057846; doi:10.1371/journal.pone.0281752)

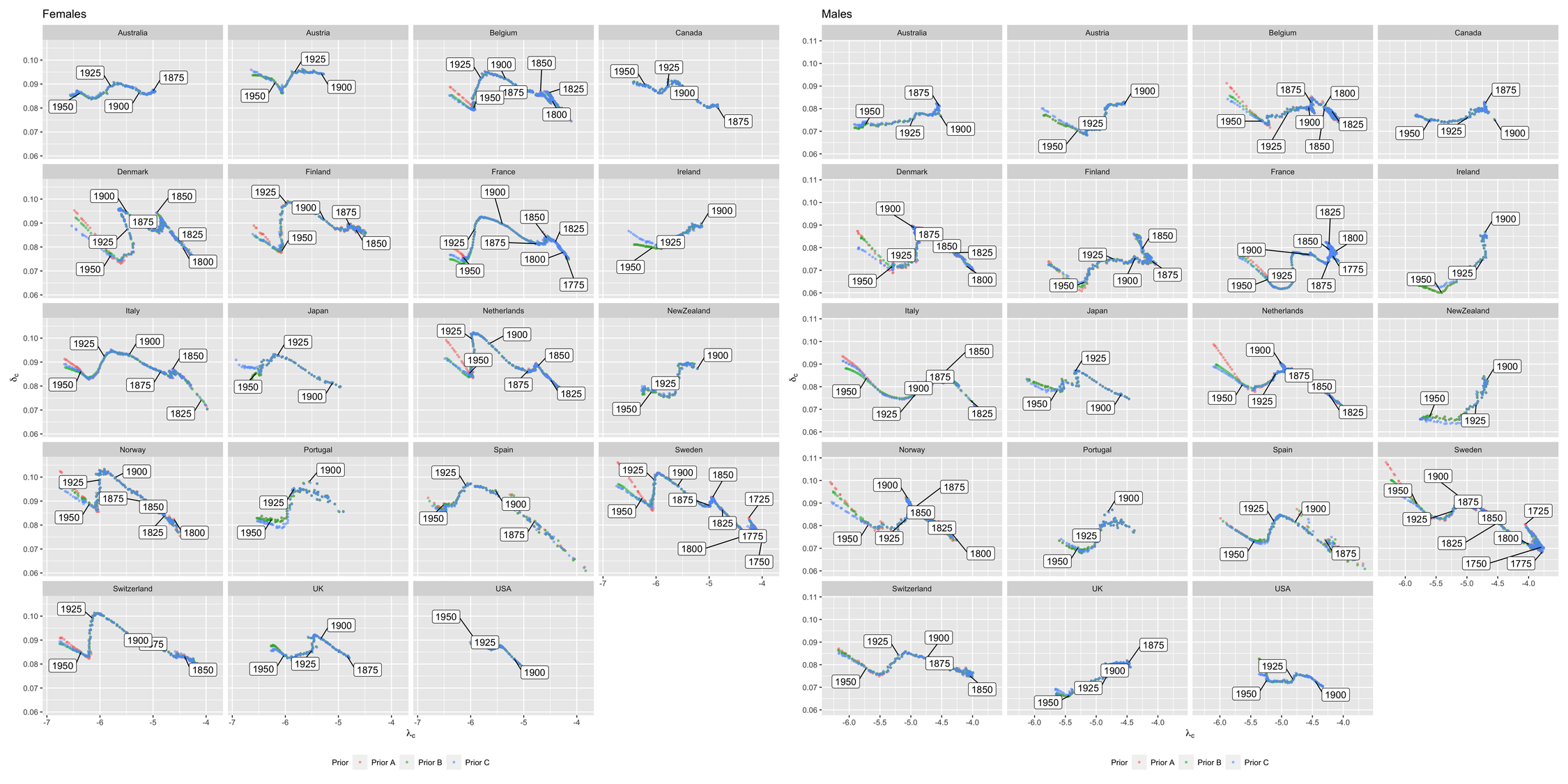

Supplement: S1 Fig — Note: The points show the modal estimate of the RDA (δc) and logged mortality rates at age 50 (λc) for each cohort. Confidence intervals are not shown to increase clarity. (TIF) [file pone.0281752.s001.tif]

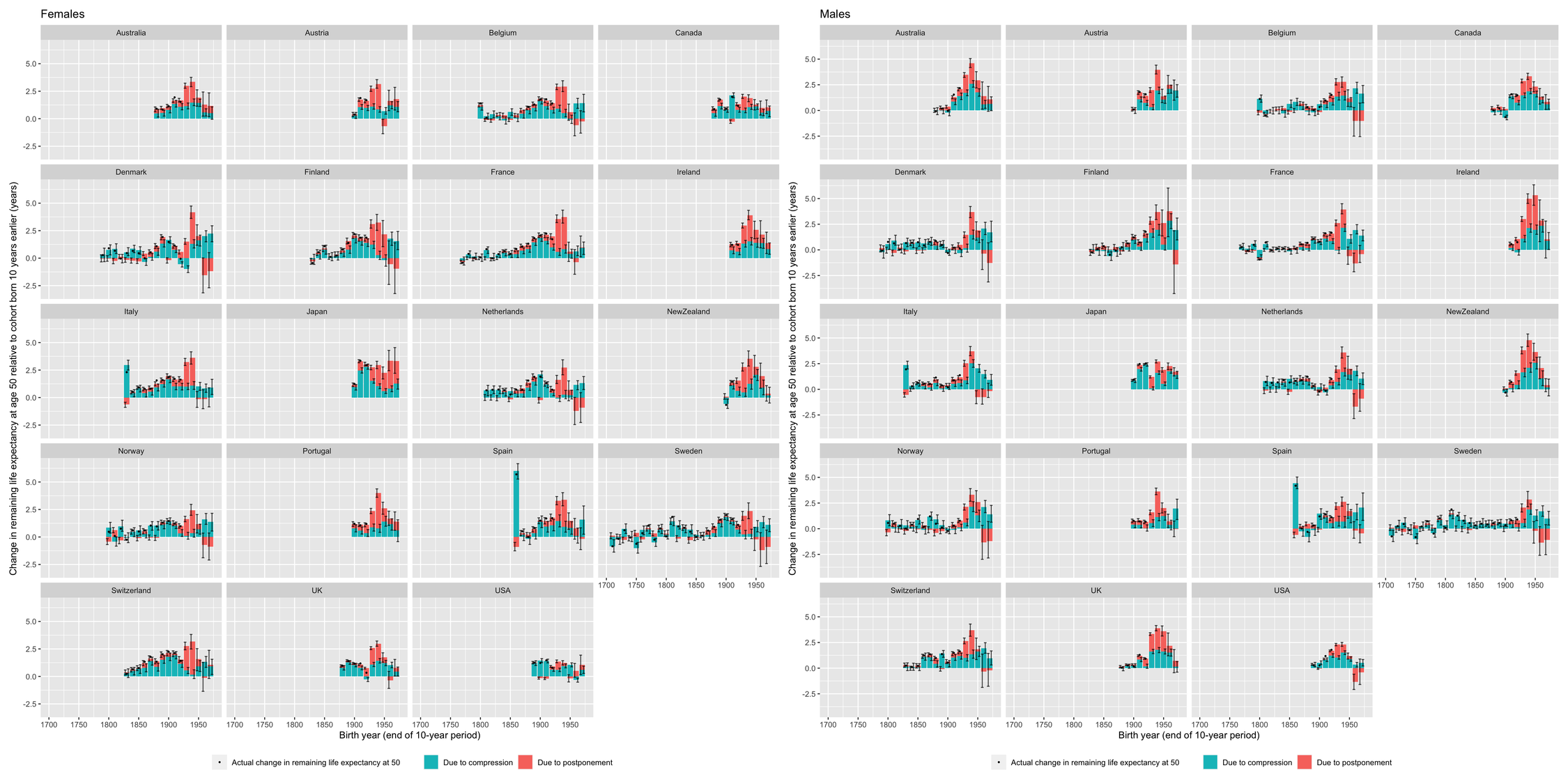

Supplement: S2 Fig — Note: The coloured bars show the median estimate of 10-year changes in remaining cohort life expectancy at age 50, divided into the portion due to postponement and the portion due to compression, determined using the methodology shown in Fig 1 of the main text and formulae derived in the methods section. Black dots represent the actual change in life expectancy for cohorts that reached the age of 100 in our data. (TIF) [file pone.0281752.s002.tif]

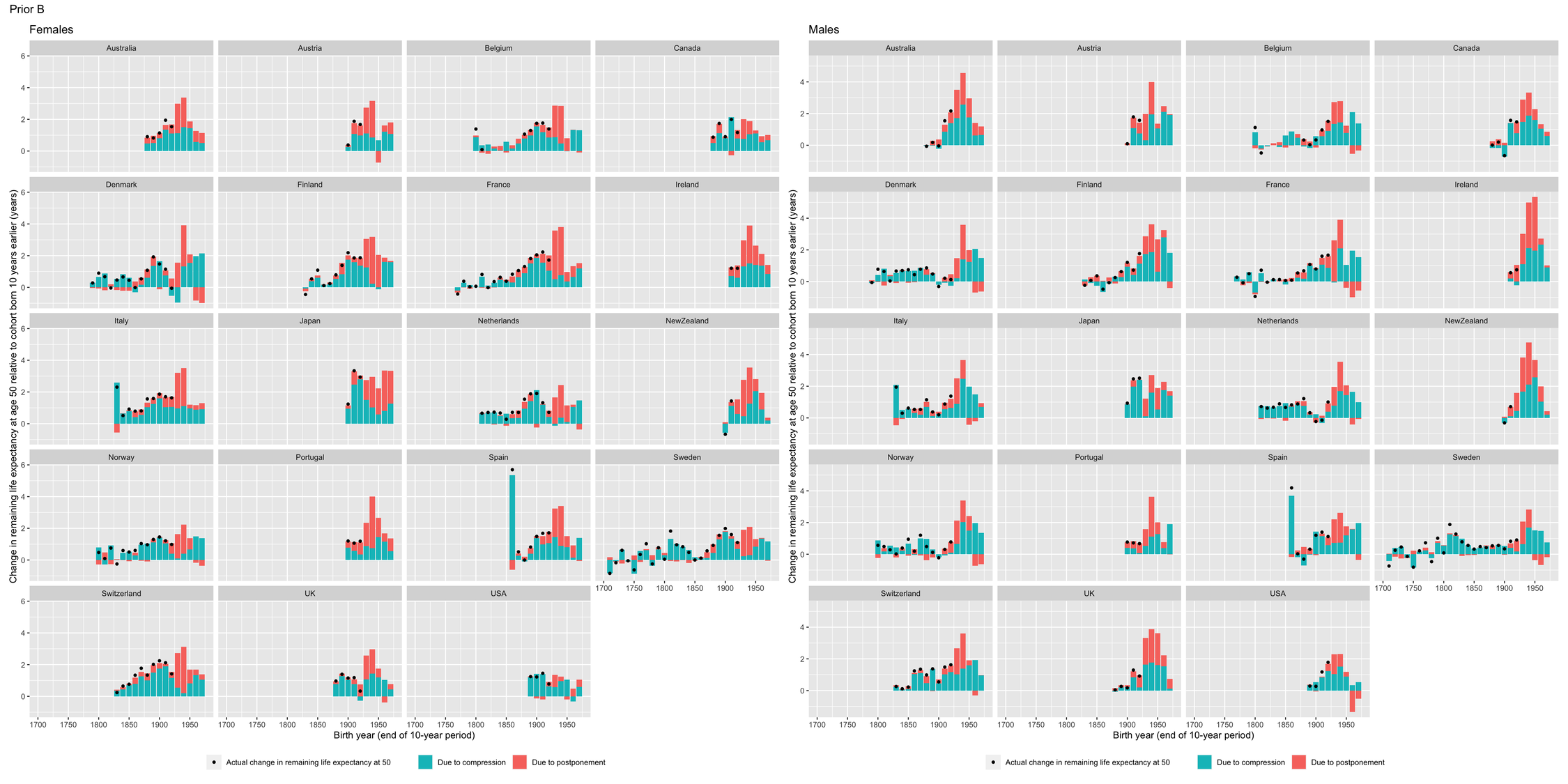

Supplement: S3 Fig — (TIF) [file pone.0281752.s003.tif]

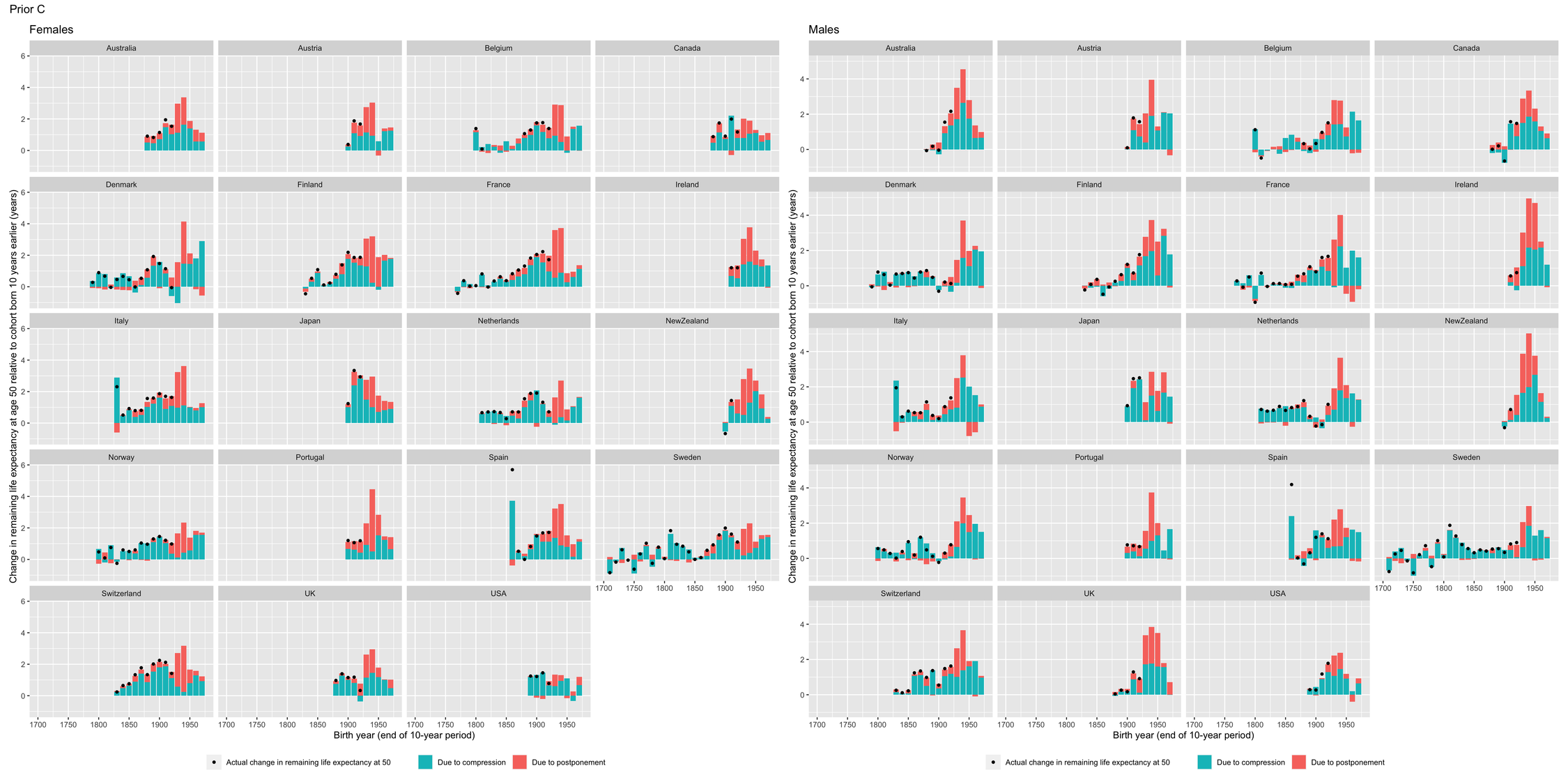

Supplement: S4 Fig — (TIF) [file pone.0281752.s004.tif]

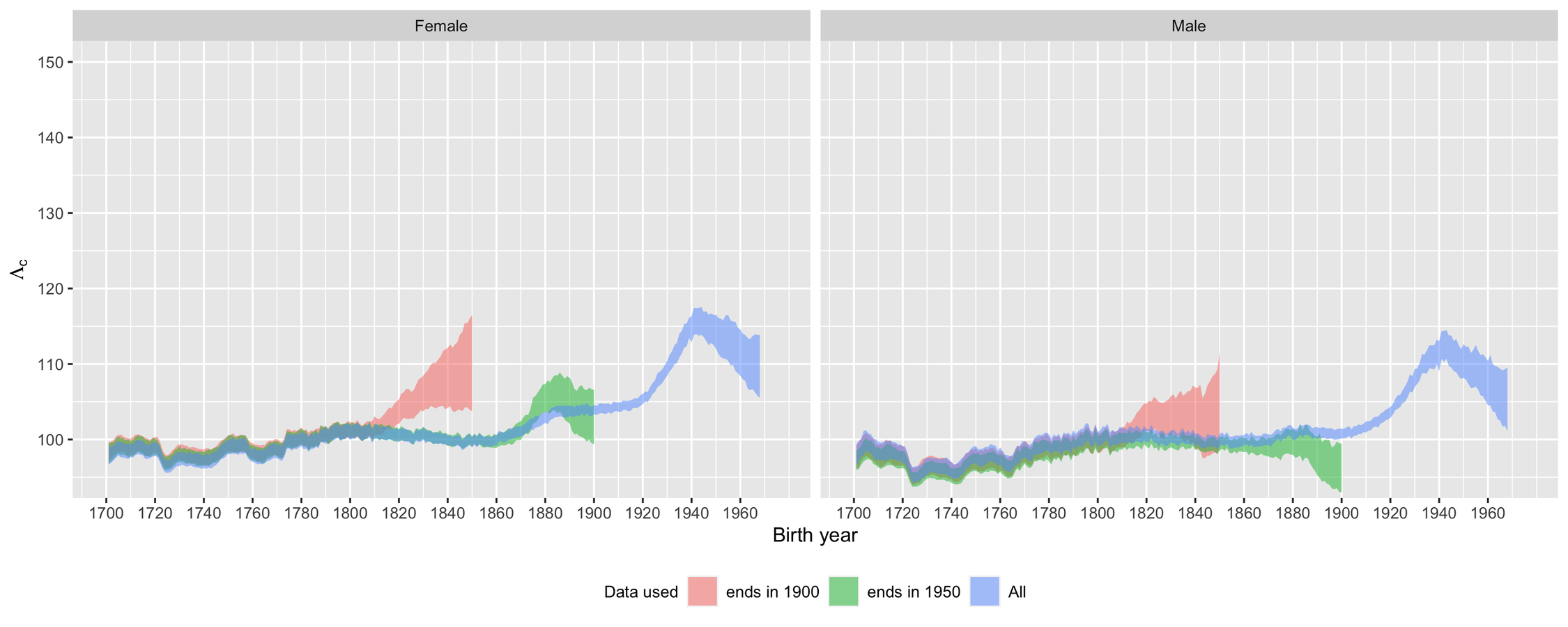

Supplement: S5 Fig — Confidence intervals for Λc data to 1900, data to 1950 and all data. (TIF) [file pone.0281752.s005.tif]

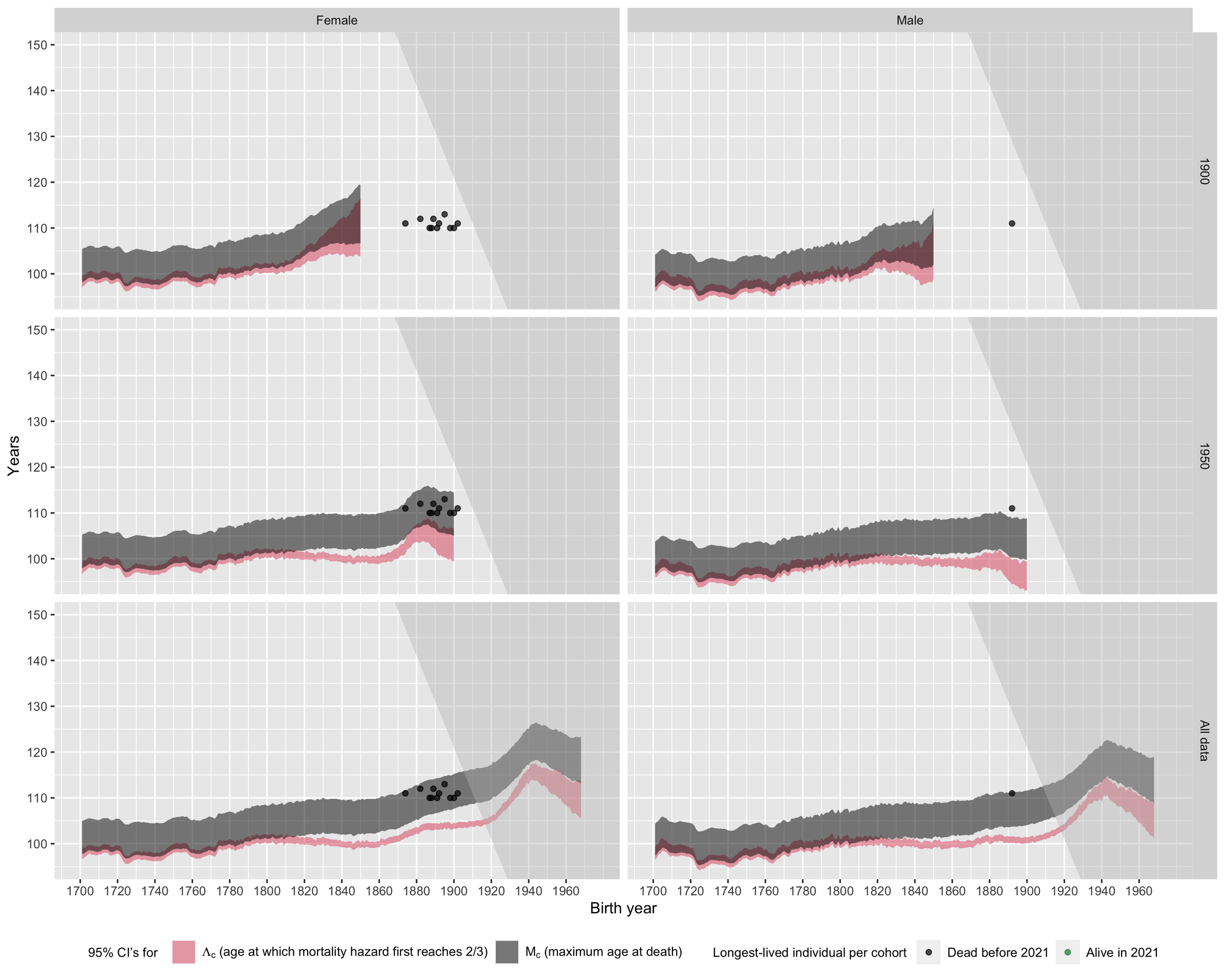

Supplement: S6 Fig — Confidence intervals for Mc using data to 1900, data to 1950 and all data. (TIF) [file pone.0281752.s006.tif]

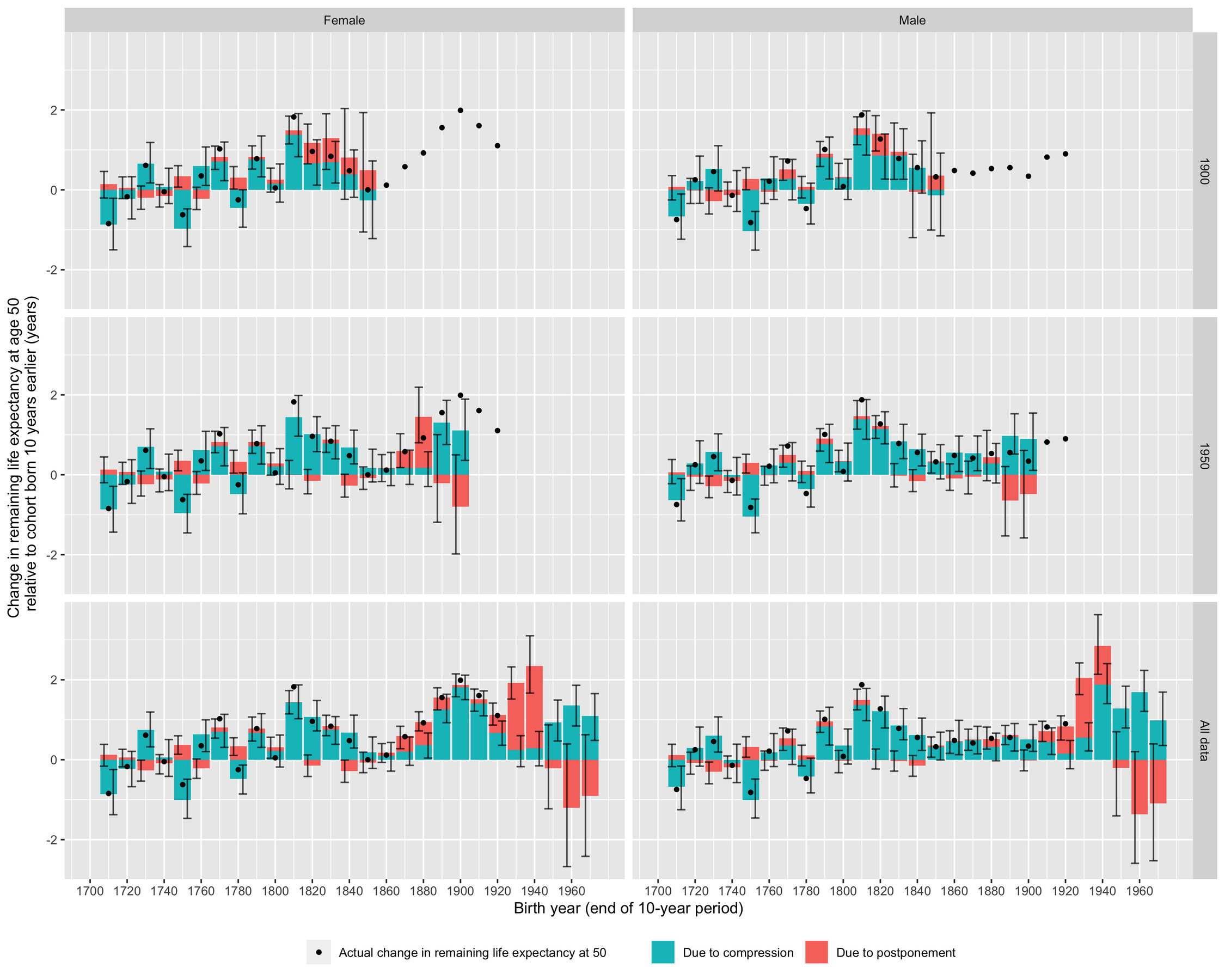

Supplement: S7 Fig — Predicted changes in base life expectancy due to compression and postponement using all data to 1900, data to 1950 and all data. (TIF) [file pone.0281752.s007.tif]

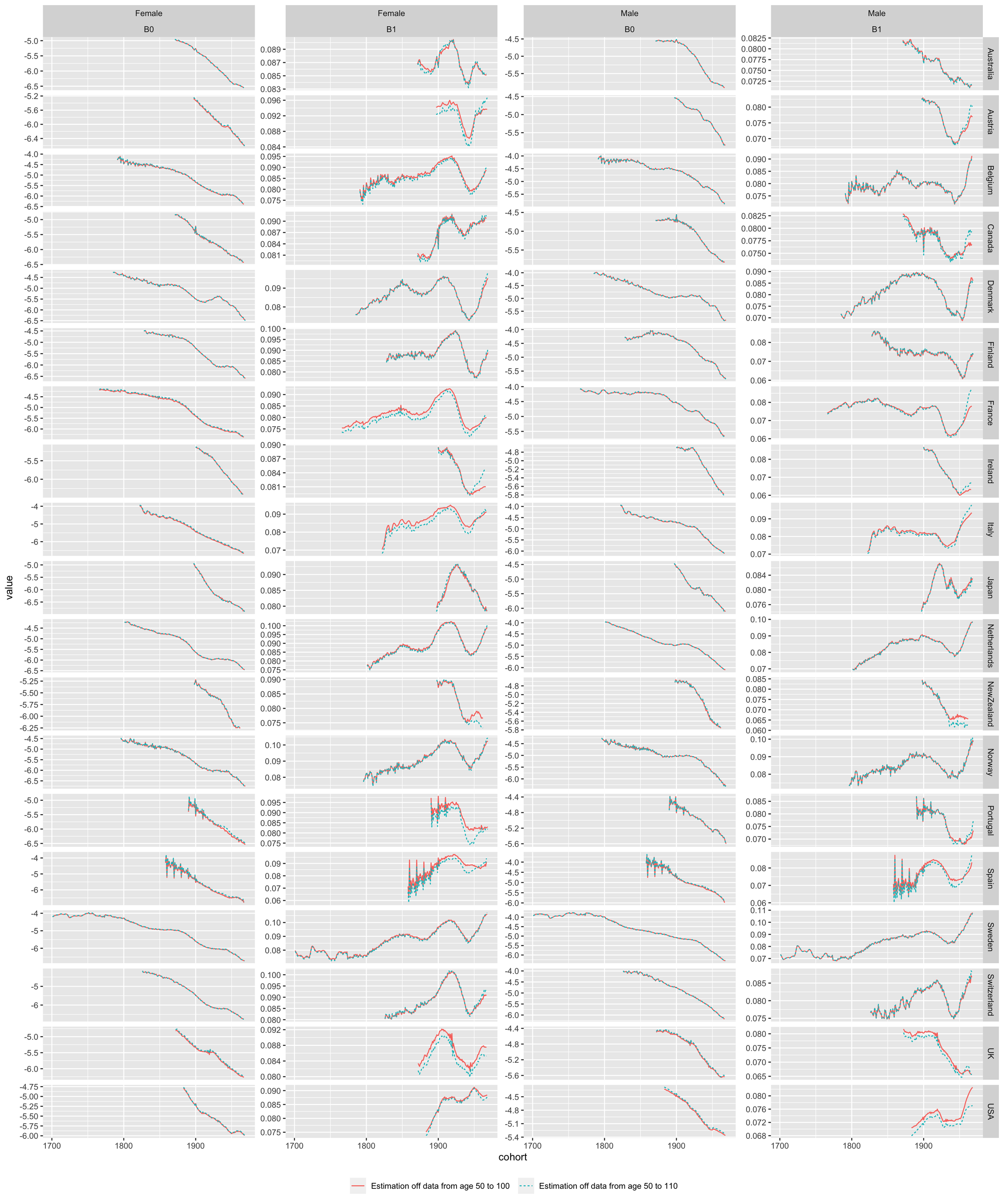

Supplement: S8 Fig — (TIF) [file pone.0281752.s008.tif]

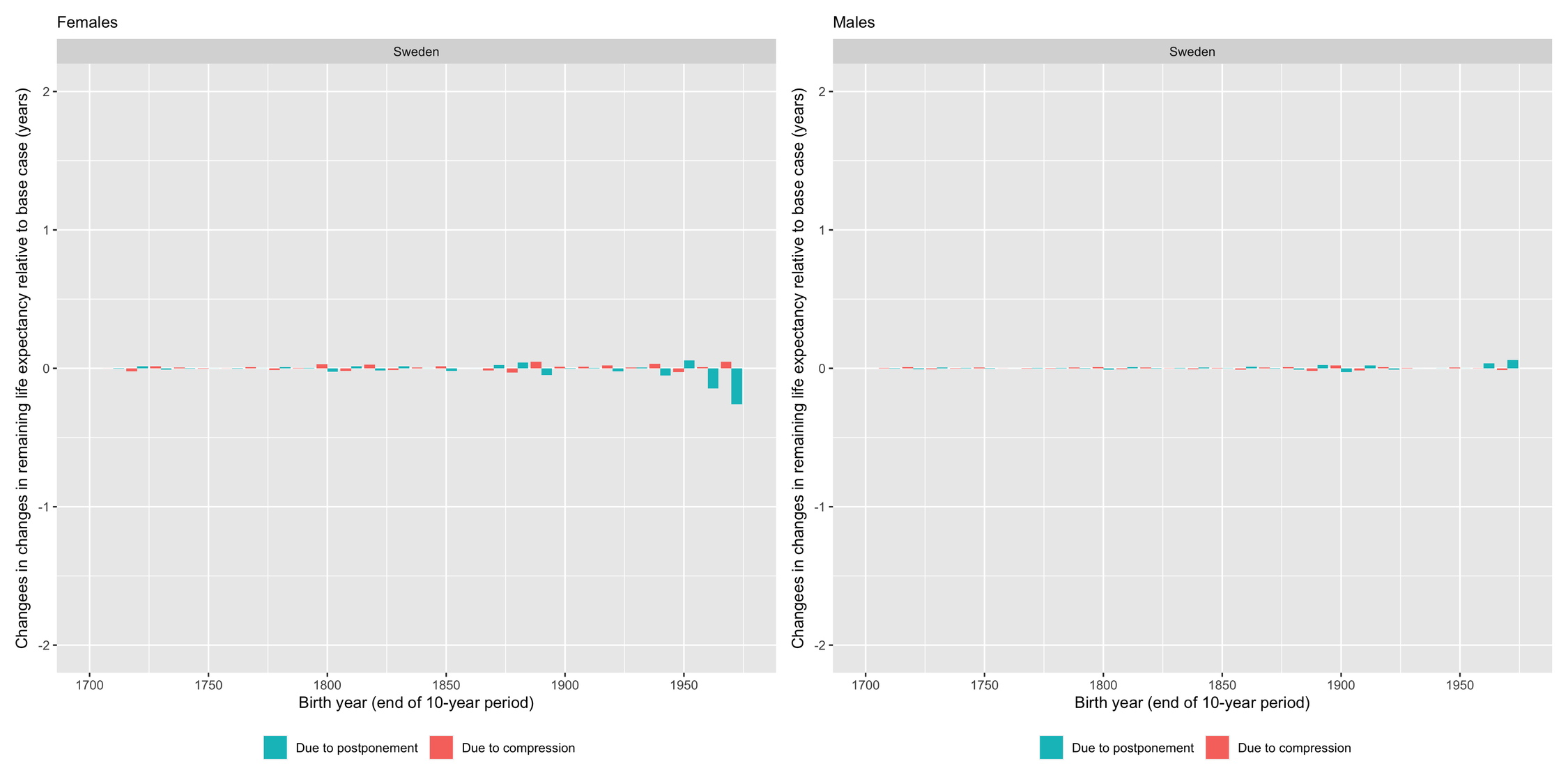

Supplement: S9 Fig — (TIF) [file pone.0281752.s009.tif]

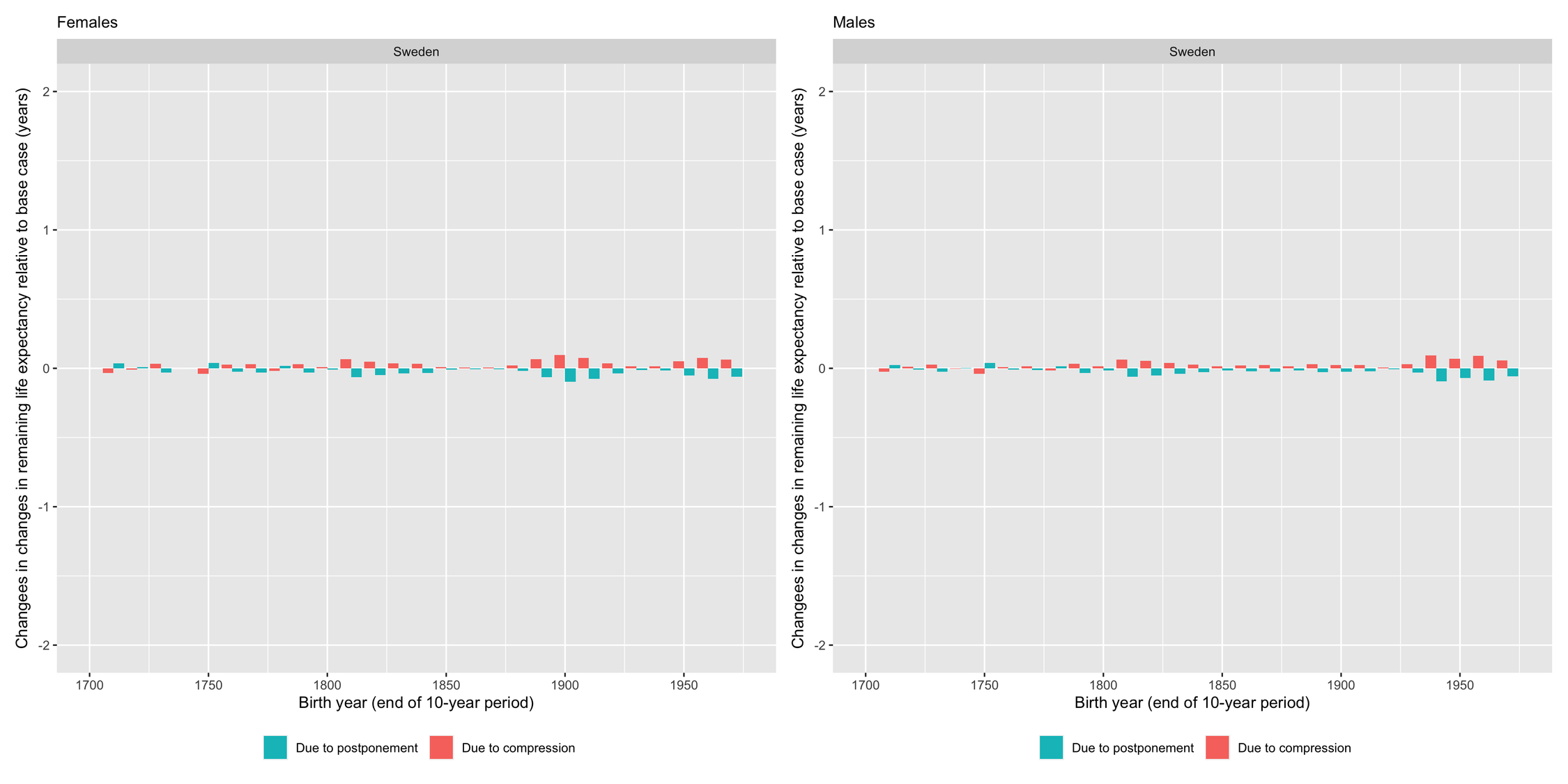

Supplement: S10 Fig — (TIF) [file pone.0281752.s010.tif]

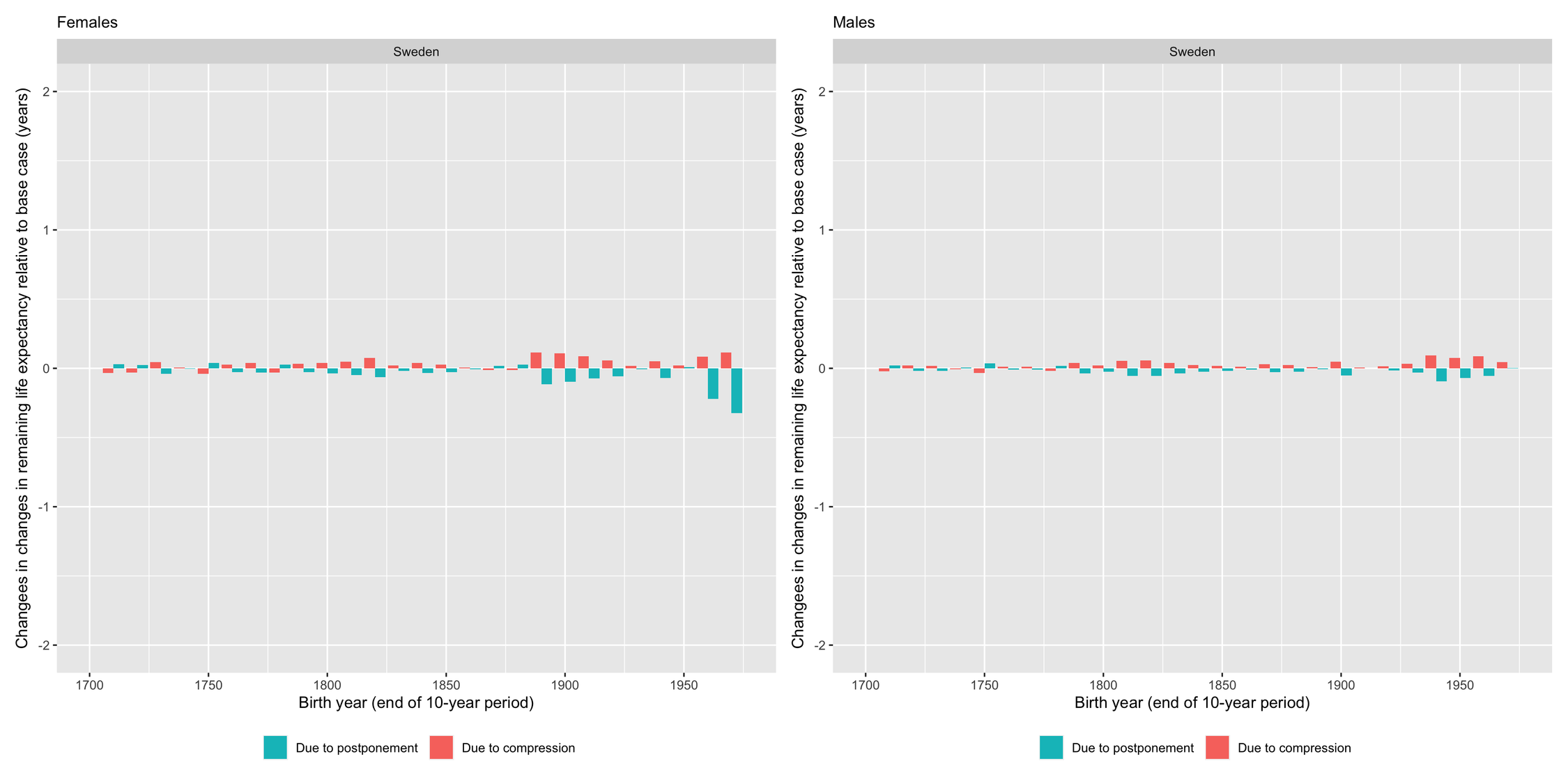

Supplement: S11 Fig — (TIF) [file pone.0281752.s011.tif]

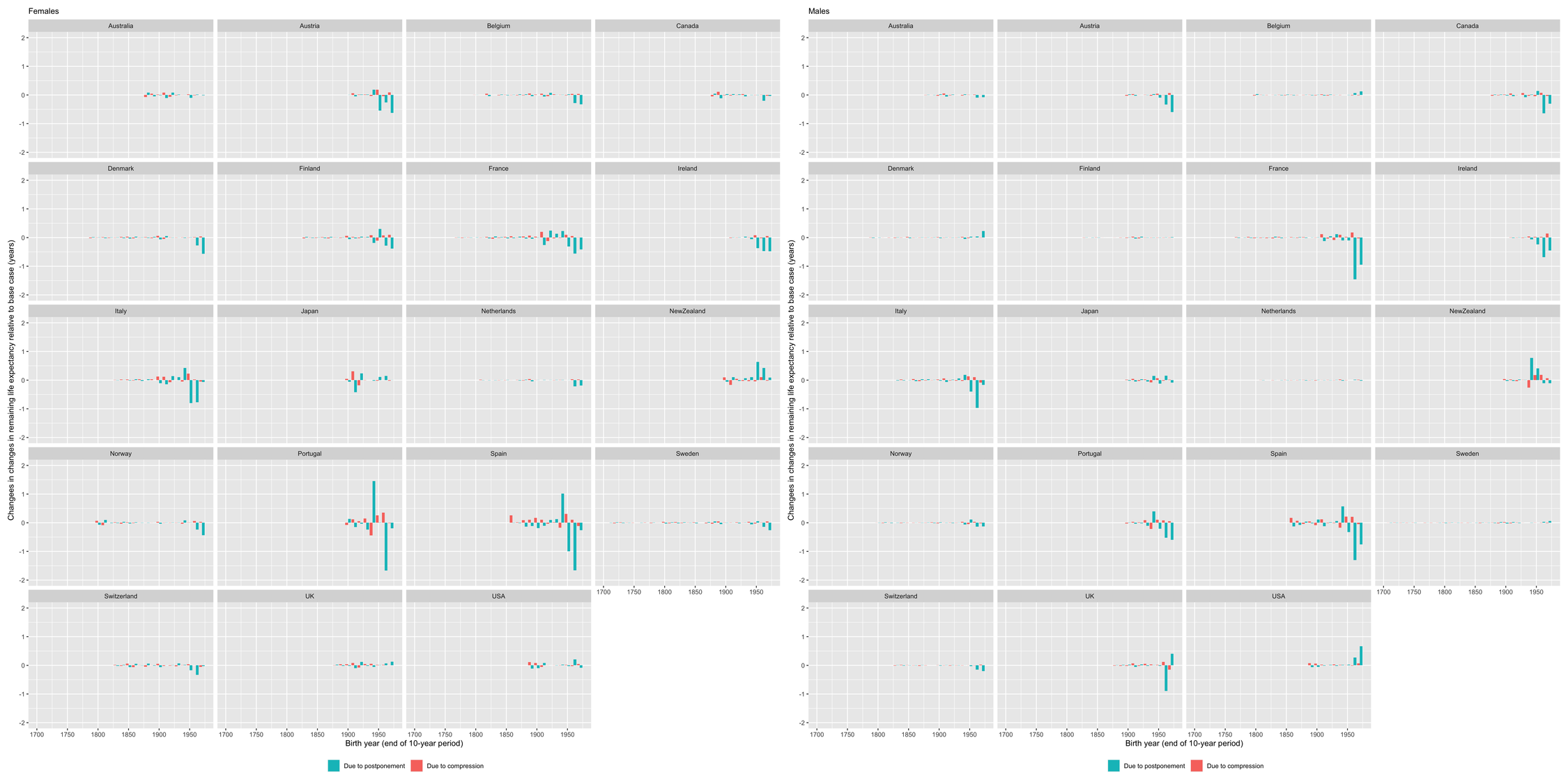

Supplement: S12 Fig — (TIF) [file pone.0281752.s012.tif]

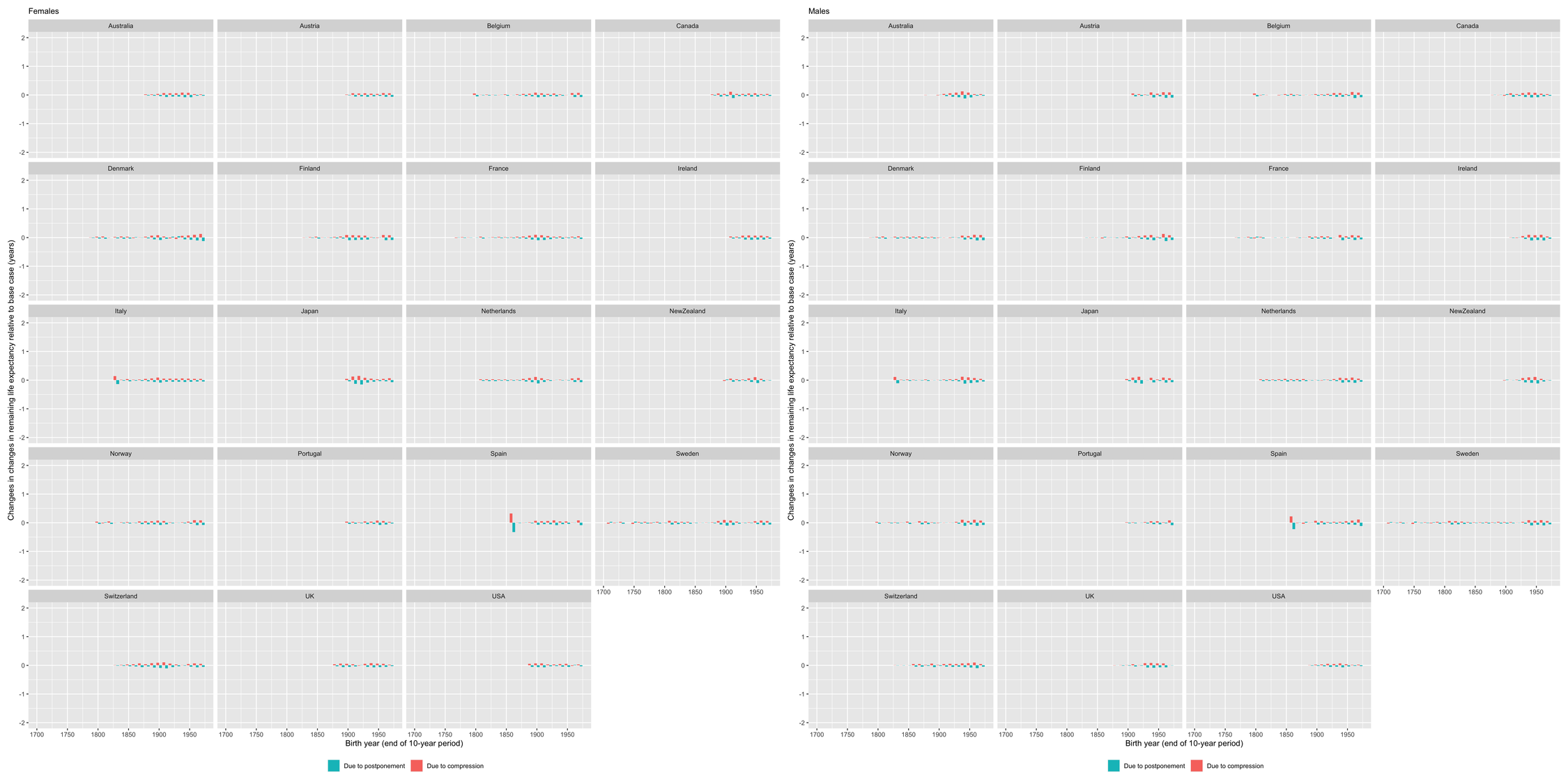

Supplement: S13 Fig — (TIF) [file pone.0281752.s013.tif]

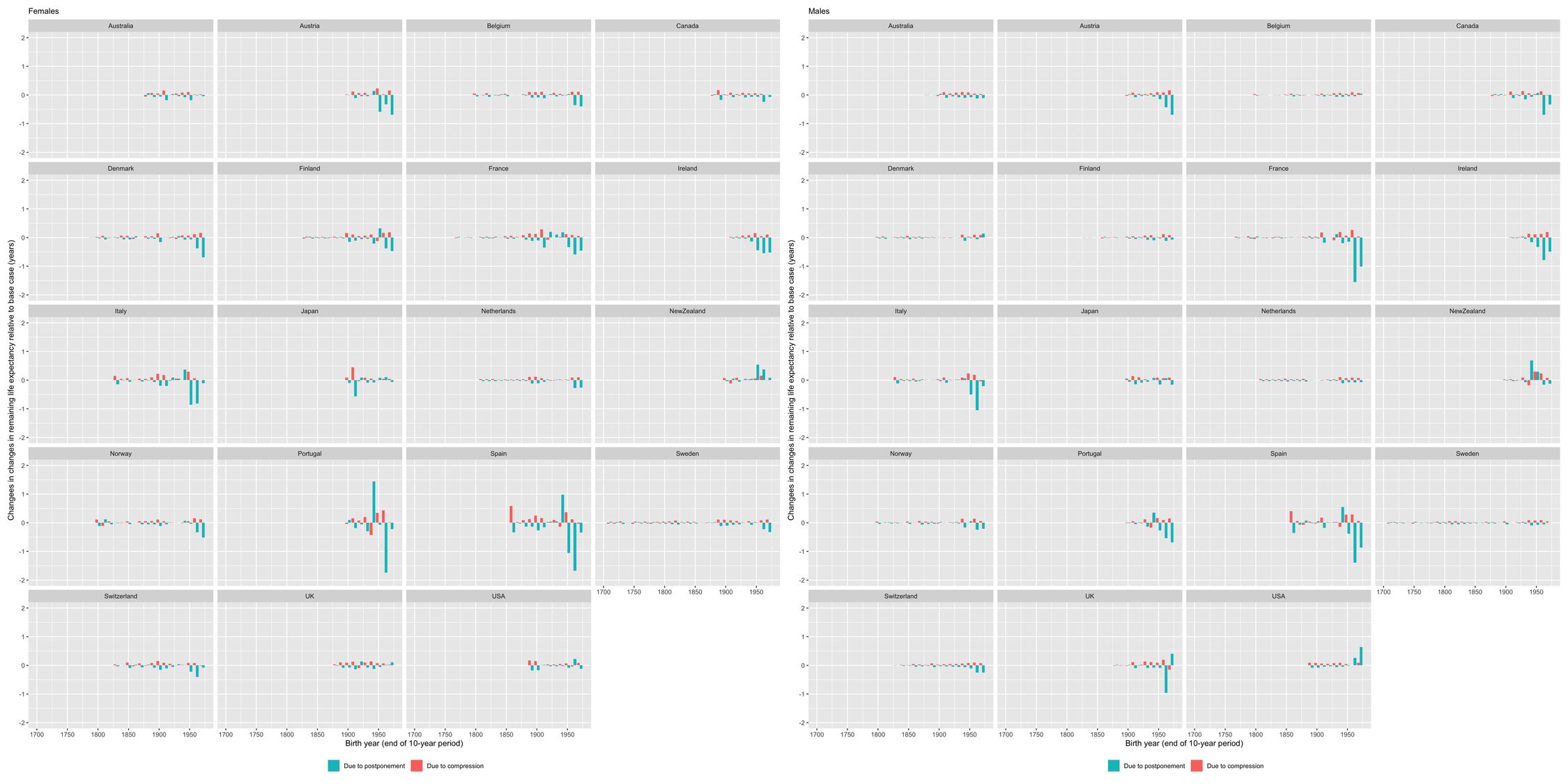

Supplement: S14 Fig — (TIF) [file pone.0281752.s014.tif]
