# Supplementary material for: Mortality postponement and compression at older ages in human cohorts
Source: PLoS One. 2023 Mar 29;18(3):e0281752. doi: 10.1371/journal.pone.0281752 (PMC10057846; doi:10.1371/journal.pone.0281752)
Supplement: S1 File — (DOCX) [file pone.0281752.s015.docx]

1. **Supporting information**

*M1. Bayesian estimation technique*

Details are available in McCarthy (2020). Assume we have N data points, so N combinations of x and c. Writing the vector of *N* error terms as $\bar{\mathbf{\varepsilon}}$, we assume that $\bar{\mathbf{\varepsilon}}\sim MVN(\mathbf{0},\mathbf{\Sigma})$, (MVN denotes the Multivariate Normal Distribution) where $\mathbf{\Sigma}=\sigma_{\varepsilon}^{2}\mathbf{I}_{\mathbf{N}}+\mathbf{I}_{\mathbf{\omega}}+\sigma_{\gamma}^{2}\mathbf{I}_{\mathbf{x}}$ . $\sigma_{\varepsilon}^{2}$ represents model misfit, $\mathbf{I}_{\mathbf{N}}$ is a size-*N* identity matrix, $\mathbf{I}_{\mathbf{\omega}}$ is an *N*x*N* matrix with all elements zero except on the diagonal, with the *i*’th diagonal element equal to $\omega_{x_{i},c_{i}}=1/D_{x_{i},c_{i}}$, where $x_{i}$ is the age of the *i*’th data point, and $c_{i}$ the year of birth. (Fay and Herriot, 1979) $\mathbf{I}_{\mathbf{x}}$ is an *N*x*N* matrix with the *ij’*th element equal to 1 if the *i*’th and *j*’th data points share the same age, that is $x_{i}=x_{j}.$ $\sigma_{\gamma}^{2}$ therefore clusters standard errors by age, to account for any age patterns in mortality that are not accounted for by the modified Gompertz law, such as any slowdown in the rate of increase in mortality with age at very old ages, referenced earlier, which might be caused, *inter alia*, by intra-cohort heterogeneity in mortality rates, and the apparent delayed onset of senescent ageing in recent cohorts of females, possibly caused by delayed parity (Shirazi et al, 2020)

To model changes in the level and slope of logged age-mortality profiles across birth cohorts, the first difference of the shape parameters b are assumed to follow a Vector Auto-Regression (VAR). Writing $\mathbf{\beta}_{\mathbf{c}}=(\lambda_{50,c},\delta_{c})$, the row vector comprising shape parameters for the age-mortality profile of cohort *c*, and $\mathbf{\beta=\{\beta}_{\mathbf{c}}\mathbf{\}}_{c=1...C}$, where *C* is the number of cohorts in the sample, the matrix of stacked cohort shape parameter vectors gives:

$(\Delta\mathbf{\beta})_{c}-\hat{\mathbf{\mu}}-\hat{\mathbf{M}}_{\mathbf{1}}(\Delta\mathbf{\beta})_{c-1}=\mathbf{\nu}_{\mathbf{c}}$, (4)

where $\Delta$ is a row-by-row (cohort-by-cohort) difference operator and $\mathbf{\nu}_{\mathbf{c}}\sim MVN(\mathbf{0},\hat{\mathbf{S}})$, $\hat{\mathbf{\mu}}$ represents the mean change in $\Delta\mathbf{\beta}$, and $\hat{\mathbf{M}}_{\mathbf{1}}$ a matrix of VAR coefficients on lagged values of $\Delta\mathbf{\beta}$ .

In the first stage, ML is used to estimate values of $\mathbf{\{\beta}_{\mathbf{c}}\mathbf{\}}_{c=...}$cohort-by-cohort as follows:

$\log(m_{x,c})=\lambda_{50,c}+\delta_{c}(x-50)+\varepsilon_{x,c}$ (5)

where $\varepsilon_{x,c}\sim N(0,\sigma_{\varepsilon}^{2}+\omega_{x,c})$. A subset of these estimates from extinct or nearly-extinct cohorts are then used to obtain estimates of the parameters $\hat{\mathbf{\mu}}\mathbf{,}\hat{\mathbf{M}}_{\mathbf{1}}\mathbf{,}\hat{\mathbf{S}}$ of the VAR in (M2) , giving a prior distribution for the 2x(*C*-1) matrix$\Delta\mathbf{\beta}$, where *C* is the number of cohorts in the sample:

, (6)

where denotes the density of a MVN distribution with known mean **b** and variance-covariance matrix **X**, evaluated at point **a**.

In the second estimation stage, Bayes’ Theorem is used to combine the prior distribution for $\Delta\mathbf{\beta}$ in (6) with the likelihood function of the data conditional on $\mathbf{\beta}$, , which is easily obtained from (2) using standard results, to obtain the posterior distribution for $\mathbf{\beta}$. The logged density of the posterior distribution of $\mathbf{\beta}$ is then:

(7)

We obtained the mode of (7) using numerical optimisation techniques. Using this mode as a starting point, we then used the Metropolis-Hastings algorithm to draw a sample of 1,000 values of $\beta$ from the posterior. We used a burn-in period of 500 draws, and thereafter selected every 99^th^ draw until we had a sample of 1,000 observations.

*M2. Division between compression and postponement*

The remaining life expectancy at age 50 of a cohort that has mortality governed by (2) in each future year, $e_{50}(\lambda_{50,c},\delta_{c})\equiv E[\tilde{e}_{50}(\lambda_{50,c},\delta_{c},\sigma^{2})]$, where $\tilde{e}_{50,c}$, the observed remaining life expectancy at age 50 of cohort c, is a random variable that depends on future realizations of $\tilde{\varepsilon}_{x,c}$. If we assume that $\tilde{\varepsilon}_{x,c}$ is constant over year of age, and writing $\left\lfloor z\right\rfloor=trunc(z)$ we have:

$\tilde{e}_{50,c}=\int_{50}^{\infty} \exp(-\int_{50}^{a} \mu_{z,c}\exp(\tilde{\varepsilon}_{\left\lfloor z\right\rfloor,c})dz)da$. (9)

$e_{50}(\lambda_{50,c},\delta_{c})$ is not analytically tractable.

We therefore define the remaining base life expectancy at age 50, called $BLE(\lambda_{50,c},\delta_{c})$, as the life expectancy of a cohort who suffers exactly the log base mortality $\log(\mu_{x,c})$ at each instant from age 50 and older (so setting $\tilde{\varepsilon}_{x,c}\equiv E[\tilde{\varepsilon}_{x,c}]=0$ in (3)). Note that $BLE(\lambda_{50,c},\delta_{c})$ is a biased estimate of $e_{50}(\lambda_{50,c},\delta_{c})$ due to convexity. However, over our range of RMSE, the bias varies between ~2% for earlier cohorts and ~0.2% for later cohorts, and so we ignore it here.

The individuals in cohort c have base mortality defined using the Gompertz Law up to age $\Lambda_{c}$ and a constant mortality hazard rate of 2/3 thereafter. After age $\Lambda_{c}$, death is therefore a Poisson process with constant intensity parameter 2/3 per year and the length of life after $\Lambda_{c}$ is exponentially distributed with the same parameter, so remaining life expectancy at $\Lambda_{c}$ is exactly 1.5 years.

The probability that an individual in cohort c aged 50 will reach age $\Lambda_{c}$ if they have mortality in each future year equal to base mortality is equal to

.

Using standard results (Lenart & Missov, 2010) base life expectancy age 50 can therefore be calculated as

(10)

where $E_{1}[z]$ is the exponential integral, defined as:

$E_{1}[z]=\int_{z}^{\infty} \frac{\exp(-y)}{y}dy$. (11)

The change in base life expectancy between two cohorts *a* and *b* with $b>a$can then be calculated as:

$BLE(\lambda_{50,b},\delta_{b})-BLE(\lambda_{50,a},\delta_{a})$. (12)

We divide this change into the portion due to compression and the portion due to postponement as illustrated in Figure 1 in the text, so:

$\underbrace{BLE(\lambda_{50,b},\delta_{b})-BLE(\gamma_{0},\gamma_{1})}_{\text{Portion due to postponement}}+\overbrace{BLE(\gamma_{0},\gamma_{1})-BLE(\lambda_{50,a},\delta_{a})}^{\text{Portion due to compression}}$, (13)

where $\gamma_{0}=\lambda_{50,b}$ and $\gamma_{1}=\delta_{a}\frac{\log(2/3)-\lambda_{50,b}}{\log(2/3)-\lambda_{50,a}}$. (14)

*M3. Confidence intervals for the GMA and the maximum length of life*

We define $M_{c}$ to be the maximum lifespan of all individuals in cohort c. We wish to calculate the distribution function for $M_{c}$, that is $\Pr(M_{c}<x)$, again conditional on $\tilde{\varepsilon}_{x,c}\equiv E[\tilde{\varepsilon}_{x,c}]=0$.

We first define $N_{\Lambda_{c},c}$ to be the number of people in cohort c who reach age $\Lambda_{c}$, so

, (15)

where is the number of people in cohort c at age 50 (ignoring migration) and $Bin(n,p)$ denotes the Binomial distribution with parameters n and p. Note that

$\Pr(M_{c}>\Lambda_{c}|\delta_{c},\lambda_{50,c})\equiv\Pr(N_{\Lambda_{c},c}>0|\delta_{c},\lambda_{50,c})$.

Then

$\begin{matrix} \Pr(M_{c}<x|\delta_{c},\lambda_{50,c})=\Pr(M_{c}<x|M_{c}<\Lambda_{c},\delta_{c},\lambda_{50,c})\Pr(M_{c}<\Lambda_{c}|\delta_{c},\lambda_{50,c}) \\ \text{ }+\Pr(M_{c}<x|M_{c}\geq\Lambda_{c},\delta_{c},\lambda_{50,c})\Pr(M_{c}\geq\Lambda_{c}|\delta_{c},\lambda_{50,c}) \\ \text{ }=\left\{\begin{matrix} \Pr(M_{c}<x|N_{\Lambda_{c},c}>0,\delta_{c},\lambda_{50,c})\Pr(N_{\Lambda_{c},c}>0|\delta_{c},\lambda_{50,c})+\Pr(N_{\Lambda_{c},c}=0|\delta_{c},\lambda_{50,c})\text{ if }x>\Lambda_{c} \\ \Pr(M_{c}<x|N_{\Lambda_{c},c}=0,\delta_{c},\lambda_{50,c})\Pr(N_{\Lambda_{c},c}=0|\delta_{c},\lambda_{50,c})\text{ otherwise } \end{matrix}\right. \end{matrix}$ (16)

If $\delta_{c}$, $\lambda_{50,c}$ and $N_{\Lambda_{c},c}>0$ are known, $M_{c}$ has distribution function:

(17)

Well-known results in extreme value theory (Mood et al, 1974) indicate that$M_{c}|N_{\Lambda_{c},c}>0,\delta_{c},\lambda_{50,c}$ has an asymptotic Gumbel distribution as $N_{\Lambda_{c},c}$ gets large, with mean approximately equal to $\Lambda_{c}+1.5\log[N_{\Lambda_{c},c}]+1.5(0.577)$ and a 95% confidence interval with approximate width 7.5 years.

In our case, $\delta_{c}$, $\lambda_{50,c}$ and $N_{\Lambda_{c},c}$ are unknown, and $N_{\Lambda_{c},c}$ may be too small to use asymptotic results, so we calculate $\Pr(M_{c}<x)$ numerically by substituting (17) into (16) and integrating over the distribution of $\delta_{c}$, $\lambda_{50,c}$ and $N_{\Lambda_{c},c}$using (15) and Monte-Carlo integration over our MH sample.[[1]](#footnote-1)

One issue that arises following this approach is that we do not know $\Pr(M_{c}<x|N_{\Lambda_{c},c}=0,\delta_{c},\lambda_{50,c})$ for $x<\Lambda_{c}$. In most cases there are sufficient numbers of individuals in each cohort that $\Pr(N_{\Lambda_{c},c}=0|\delta_{c},\lambda_{50,c})\approx0$, so $M_{c}>\Lambda_{c}$ almost certainly. We therefore calculate and present $\Pr(M_{c}<x| N_{\Lambda_{c},c}>0)$. For smaller countries and in earlier cohorts, especially for males, this may overstate lower confidence intervals where $\Pr(N_{\Lambda_{c},c}=0|\delta_{c},\lambda_{50,c})>\sim0.01$.

*S1: Supplementary results*

Figure S1: Bayesian estimates of slope and intercept parameters of male and female cohorts for all countries

Note: The points show the modal estimate of the RDA ($\delta_{c}$) and logged mortality rates at age 50 ($\lambda_{c}$) for each cohort. Confidence intervals are not shown to increase clarity.

Figure S2: Implied changes in remaining cohort base life expectancy at age 50 over ten-year birth cohorts, divided into the portion due to postponement and portion due to compression

Note: The coloured bars show the median estimate of 10-year changes in remaining cohort life expectancy at age 50, divided into the portion due to postponement and the portion due to compression, determined using the methodology shown in Figure 1 of the main text and formulae derived in the methods section. Black dots represent the actual change in life expectancy for cohorts that reached the age of 100 in our data.

Figure S3: Results for Prior B

Figure S4: Results for Prior C Figure S5: Backtesting for Sweden. Confidence intervals for $\Lambda_{c}$ data to 1900, data to 1950 and all data

Figure S6: Backtesting for Sweden. Confidence intervals for $M_{c}$ using data to 1900, data to 1950 and all data

Figure S7: Backtesting for Sweden. Predicted changes in base life expectancy due to compression and postponement using all data to 1900, data to 1950 and all data

1. This approach is similar to, but more accurate than, the approximation used by Barbi et al (2003) in extinct cohorts of French data. Our Bayesian approach permits estimation (with confidence intervals) for incomplete cohorts. [↑](#footnote-ref-1)
