# Supplementary material for: Mortality postponement and compression at older ages in human cohorts
Source: PLoS One. 2023 Mar 29;18(3):e0281752. doi: 10.1371/journal.pone.0281752 (PMC10057846; doi:10.1371/journal.pone.0281752)
Supplement: S2 File — (DOCX) [file pone.0281752.s016.docx]

More information – including results for each country – can be found at <http://www.davidgmccarthy.com/mortality-in-human-cohorts-supplementary-data>
